# Runx1 Deficiency Protects Against Adverse Cardiac Remodeling After Myocardial Infarction

**DOI:** 10.1161/CIRCULATIONAHA.117.028911

**Published:** 2017-12-26

**Authors:** Charlotte S. McCarroll, Weihong He, Kirsty Foote, Ashley Bradley, Karen Mcglynn, Francesca Vidler, Colin Nixon, Katrin Nather, Caroline Fattah, Alexandra Riddell, Peter Bowman, Elspeth B. Elliott, Margaret Bell, Catherine Hawksby, Scott M. MacKenzie, Liam J. Morrison, Anne Terry, Karen Blyth, Godfrey L. Smith, Martin W. McBride, Thomas Kubin, Thomas Braun, Stuart A. Nicklin, Ewan R. Cameron, Christopher M. Loughrey

**Affiliations:** 1 Glasgow Cardiovascular Research Centre, Institute of Cardiovascular and Medical Sciences, University of Glasgow, University Place, UK (C.S.M., W.H., A.B., K.M., F.V., K.N., C.F., A.R., P.B., E.B.E., C.H., S.M.M., G.L.S., M.W.M., S.A.N., C.M.L.); 2 Division of Cardiovascular Medicine, Addenbrooke’s Centre for Clinical Investigation, University of Cambridge, Addenbrooke’s Hospital, UK (K.F.); 3 School of Veterinary Medicine (M.B., E.R.C.); 4 Centre for Virus Research (A.T.), University of Glasgow, Garscube Campus, UK; 5 Division of Infection and Immunity, The Roslin Institute, University of Edinburgh, Easter Bush, Midlothian, UK (L.J.M.); 6 Cancer Research UK Beatson Institute, Bearsden, Glasgow, UK (C.N., K.B.); 7 Cardiac Development and Remodeling, Max-Planck-Institute for Heart and Lung Research, Bad Nauheim, Germany (T.K., T.B.).

**Keywords:** calcium, cardiac remodeling, ventricular, myocardial infarction, myocytes, cardiac, sarcoplasmic reticulum

## Abstract

**Background:**

Myocardial infarction (MI) is a leading cause of heart failure and death worldwide. Preservation of contractile function and protection against adverse changes in ventricular architecture (cardiac remodeling) are key factors to limiting progression of this condition to heart failure. Consequently, new therapeutic targets are urgently required to achieve this aim. Expression of the Runx1 transcription factor is increased in adult cardiomyocytes after MI; however, the functional role of Runx1 in the heart is unknown.

**Methods:**

To address this question, we have generated a novel tamoxifen-inducible cardiomyocyte-specific *Runx1*-deficient mouse. Mice were subjected to MI by means of coronary artery ligation. Cardiac remodeling and contractile function were assessed extensively at the whole-heart, cardiomyocyte, and molecular levels.

**Results:**

*Runx1*-deficient mice were protected against adverse cardiac remodeling after MI, maintaining ventricular wall thickness and contractile function. Furthermore, these mice lacked eccentric hypertrophy, and their cardiomyocytes exhibited markedly improved calcium handling. At the mechanistic level, these effects were achieved through increased phosphorylation of phospholamban by protein kinase A and relief of sarco/endoplasmic reticulum Ca^2+^-ATPase inhibition. Enhanced sarco/endoplasmic reticulum Ca^2+^-ATPase activity in Runx1-deficient mice increased sarcoplasmic reticulum calcium content and sarcoplasmic reticulum–mediated calcium release, preserving cardiomyocyte contraction after MI.

**Conclusions:**

Our data identified Runx1 as a novel therapeutic target with translational potential to counteract the effects of adverse cardiac remodeling, thereby improving survival and quality of life among patients with MI.

## Abstract 2

Supplemental Digital Content is available in the text.

**Clinical Perspective**

**What Is New?**

- Our study provides new evidence that *Runx1*, a gene intensively studied in the cancer and blood research fields, has a critical role in cardiomyocytes after myocardial infarction.
- We provide conclusive evidence that increased Runx1 expression under pathological conditions leads to decreased cardiac contractile function.
- Experiments performed with a newly generated cardiomyocyte-specific Runx1-deficient mouse reveal that reducing Runx1 function preserves myocardial contractility and prevents adverse cardiac remodeling after myocardial infarction.

**What Are the Clinical Implications?**

- Our mechanistic data robustly demonstrate that Runx1 modulates cardiac sarcoplasmic reticulum calcium uptake and contractile function.
- Reducing Runx1 function drives increased contractility after myocardial infarction, thereby preserving left ventricular systolic function and preventing adverse cardiac remodeling.
- Our study therefore identifies Runx1 as a new target holding major promise for limiting the progression to heart failure among patients with myocardial infarction by preventing adverse cardiac remodeling.

Acute coronary artery blockage leading to prolonged ischemia and subsequent cardiomyocyte death (myocardial infarction [MI]) initiates a reparative process in the heart that is associated with the generation of regional infarct tissue composed predominately of fibrillar collagens. The surviving cardiomyocytes undergo eccentric hypertrophy, a process characterized by cardiomyocyte elongation with reduced diameter and impaired calcium handling, in particular decreased sarcoplasmic reticulum (SR)–mediated calcium uptake.^1^ These cellular changes are fundamental to adverse cardiac remodeling, which manifests clinically as left ventricular (LV) wall thinning, dilation, and reduced contractility.^2^ Together with neurohumoral activation, adverse cardiac remodeling after MI leads to the clinical syndrome of systolic heart failure, which, despite optimized medical therapy, is associated with extremely high mortality rates.^3^ Novel therapeutic strategies to preserve LV contractile function and to limit adverse cardiac remodeling are therefore urgently required to treat patients with MI and to improve survival rates and quality of life.

The Runx gene family (*RUNX1, RUNX2*, and *RUNX3*) encodes DNA-binding α subunits that partner core binding factor β to form heterodimeric transcription factors.^4^ RUNX proteins act as both activators and repressors of target genes in normal development and disease states.^4^ To date, most research has focused on the role of *RUNX1* in hematopoiesis owing to the frequent involvement of this gene in leukemic translocations.^4^ In contrast, little was known about the role of Runx1 in the heart. This discrepancy is not surprising given that although Runx1 expression is reported in neonatal cardiomyocytes, it decreases to minimal levels in adult cardiomyocytes.^5,6^ However, studies have demonstrated that Runx1 is reactivated in cardiomyocytes of the border zone (BZ) region adjacent to the infarct in both patients with MI and experimental animal models.^5,7^ Whether activation of Runx1 in adult cardiomyocytes after MI is simply a marker of myocardial damage or actually plays a role in the progression of adverse cardiac remodeling is currently unknown.

We have now addressed this question by inducing MI in a mouse model in which *Runx1* has been specifically excised in cardiomyocytes. We report that these mice were protected against adverse cardiac remodeling after MI, with markedly preserved LV systolic function through improved SR-mediated calcium uptake. Reactivation of Runx1 after MI therefore plays a crucial role in excitation-contraction coupling and adverse cardiac remodeling and represents a new therapeutic target with the potential to limit progression to heart failure among patients with MI.

## Methods

Detailed methods are provided in the online-only Data Supplement. The care and use of animals were in accordance with the UK Government Animals (Scientific Procedures) Act 1986. All animal procedures were approved by the University of Glasgow Animal Welfare and Ethical Review Body and licensed by the Home Office, UK (project license no. 600/4503).

### Coronary Artery Ligation

Mice 10 to 12 weeks of age (weight, 25–30 g) underwent thoracotomy and left anterior descending coronary artery ligation (permanent/temporary) using standard approaches.

### Generation of Cardiomyocyte-Specific Runx1-Knockout Mice

*Runx1*^*fl/fl*^ mice, described previously,^8^ were crossed with mice expressing tamoxifen-inducible Cre recombinase (*MerCreMer*) under the control of the cardiac-specific *αMHC* (α-myosin heavy chain)^9^ to produce the relevant test and control cohorts (online-only Data Supplement). Polymerase chain reaction (PCR) of genomic DNA, RNA isolation, cDNA synthesis, real-time quantitative PCR analysis, and immunoblotting are detailed in the online-only Data Supplement.

### Cardiac Phenotyping

Echocardiographic M-mode measurements were performed before and after left anterior descending coronary artery ligation and pressure-volume (PV) loop measurements recorded as a terminal procedure with the Scisense/Transonic small animal model PV system.

### Histology

Quantification of regional areas and infarct size was performed on Picrosirius Red/triphenyl tetrazolium chloride–stained histological sections with Image J and Adobe Photoshop. Cardiomyocyte size was assessed by AlexaFluor-conjugated wheat germ agglutinin (Invitrogen, UK) on adjacent sections. RNAscope with probes to specifically identify cardiomyocyte nuclei (pericentriolar material 1) and Runx1 was performed as detailed in the online-only Data Supplement. For each heart, positive (PPIB and POLR2A) and negative controls (bacterial dapB) were run (Figure I in the online-only Data Supplement).

### Calcium Measurements

Cardiomyocytes were isolated as previously described,^10^ loaded with a calcium-sensitive fluorophore (5.0 μmol/L Fura-4F AM, Invitrogen), and perfused during field stimulation (1.0 Hz, 2.0-ms duration, stimulation voltage set to 1.5 times the threshold). The Fura-4F fluorescence ratio (340/380-nm excitation) was measured with a spinning wheel spectrophotometer (Cairn Research Ltd; sampling rate of 5.0 kHz) to measure the cardiomyocyte intracellular calcium concentration ([Ca^2+^]_i_). Cell-edge detection (IonOptix) was used to measure cell length. Data were analyzed offline as previously described.^11^ Particular experiments used pretreatment (30 minutes) and perfusion with the protein kinase A (PKA) inhibitor H89 (1 μmol/L; Tocris Biosciences, Bristol, UK) as previously described.^12^

### Adenoviral Overexpression of *Runx1* In Vitro

Adenoviral vectors expressing either enhanced green fluorescent protein or green fluorescent protein and Runx1 in a bicistronic configuration (Ad-Runx1) were prepared and titered (online-only Data Supplement). Cardiomyocytes isolated from adult New Zealand white rabbits (3 kg) were cultured and transduced at a multiplicity of infection of 100 for 24 hours.

### Statistics

Data were expressed as mean±SEM. Comparisons between MI and sham hearts were performed with the Student *t* test on raw data before normalization to percentage change. Comparisons between >2 groups were conducted on raw data with ANOVA. In cases when the 2 control groups were combined, statistics were performed on the pooled raw data of both control groups and compared with *Runx1*^*Δ/Δ*^ mice with the Student *t* test. In experiments in which multiple isolated cardiomyocyte observations (n) were obtained from each heart (N), we have first ensured normality of data distribution and then determined the differences between control and experimental mice using linear mixed modeling (IBM SPSS Statistics, version 22) as previously published.^13^

## Results

### Expression of Runx1 After MI

Although Runx1 expression has previously been shown to increase at 3 weeks after MI, it was unknown whether increased Runx1 expression occurred at a later time point after MI (eg, 8 weeks after MI). Furthermore, temporal changes in regional Runx1 expression have not previously been investigated. Runx1 expression was therefore quantified in hearts taken from C57BL/6J mice with MI induced by permanent coronary artery ligation and compared with C57BL/6J mice that had a sham procedure but no coronary artery ligation. PV loop measurements confirmed that C57BL/6J mice with MI had reduced systolic (Figure IIA–IIC in the online-only Data Supplement) and diastolic (Figure IID and IIE in the online-only Data Supplement) function with a lower ejection fraction (Figure IIF–IIH in the online-only Data Supplement).

#### Runx1 mRNA and Protein Levels

The levels of *Runx1* mRNA increased by 2.5-fold in whole hearts 4 weeks after MI relative to 4-week sham hearts (*P*<0.05; Figure II in the online-only Data Supplement). To determine the contribution of specific myocardial regions to the observed increase in *Runx1* mRNA level, a separate cohort of sham and MI hearts was isolated, and tissue was isolated from 4 different regions (Figure IIJ in the online-only Data Supplement). *Runx1* mRNA levels analyzed with the relative quantification method increased by 5.1-fold and 1.8-fold in the infarct and BZ regions of 4-week post-MI hearts relative to the respective right ventricular (RV) region (*P*<0.05; Figure IIK in the online-only Data Supplement). No detectable change was noted in the *Runx1* mRNA levels in the LV region at 4 weeks after MI, and no statistically significant regional differences were detected in the sham hearts (Figure IIK in the online-only Data Supplement).

The levels of Runx1 protein changed in line with levels of *Runx1* mRNA (Figure IIL and IIM in the online-only Data Supplement). Runx1 protein levels increased by 6.4-fold and 13.0-fold in the BZ and infarct regions, respectively, relative to the LV region in 3-week post-MI hearts (*P*<0.05).

The pattern of *Runx1* mRNA expression was similar in 8-week post-MI hearts, with an increase of 3.7-fold and 2.2-fold in the infarct and BZ regions, respectively, relative to the corresponding RV region (*P*<0.05; Figure III in the online-only Data Supplement). However, in contrast to 4-week post-MI hearts, the *Runx1* mRNA levels were increased by 2.7-fold in the LV region relative to the RV region (*P*<0.05) at the 8-week time point (Figure III in the online-only Data Supplement).

All of the observed regional changes in *Runx1* mRNA expression occurred in the absence of any such changes in the RV region of MI hearts relative to sham hearts.

### Expression of Runx1 in Cardiomyocytes After MI

**Figure 1.**
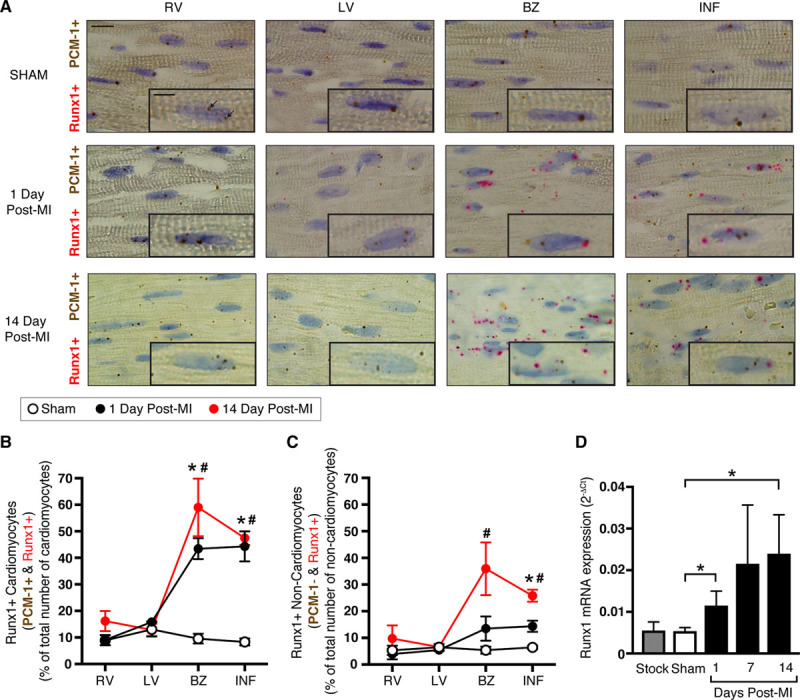
**Runx1 expression in wild-type (WT) C57BL/6 mice after myocardial infarction (MI).** **A**, Typical images of regional heart sections with RNA in situ hybridization (using RNAscope). Regions examined were the right ventricle (RV), left ventricle (LV), border zone (BZ), and infarct (INF) at 1 day (middle; n=3) and 14 days (bottom; n=3) after MI and equivalent regions in sham hearts (top; n=5). Probes for Runx1 (pink) and pericentriolar material 1 (PCM-1; cardiomyocyte-specific; brown) were used; colored punctate dots represent positive staining (arrows). Scale bar, 10 μm; magnified inset image, 5 μm. **B**, Mean quantification of cardiomyocytes (PCM-1+) and **(C)** noncardiomyocytes (PCM-1−) with Runx1-positive staining as a percent of the total number of cardiomyocytes or noncardiomyocytes, respectively. *P<0.05, 1 day after MI vs. sham. #*P*<0.05, 14 days after MI vs. sham, Student *t* test. **D**, Runx1 expression as measured by real-time quantitative polymerase chain reaction in cardiomyocytes isolated from whole sham (n=17) and 1-day (n=8), 7-day (n=6), and 14-day (n=3) post-MI hearts (ANOVA). Stock (C57BL/6J; n=4) mice were included to show that there was no detectable difference from sham hearts (ANOVA).

We next delineated the spatial-temporal expression of *Runx1* in cardiomyocytes from other cardiac cell types within different regions of the heart using RNAscope. Runx1 expression was found in 8% to 13% of cardiomyocytes and 5% to 7% of noncardiomyocytes in the 4 regions (RV, LV, and equivalent BZ and infarct zone) of sham hearts (Figure 1A–1C). Runx1 expression did not significantly differ from sham levels in the RV and LV regions at 1 and 14 days after MI (Figure 1A–1C). However, Runx1 expression significantly increased to 43% and 44% of cardiomyocytes within the BZ and infarct regions, respectively, at 1 day after MI, a time when whole-heart contractile dysfunction was also first observed (Figure 1A and 1B and Figure IV in the online-only Data Supplement). Furthermore, Runx1 expression significantly increased to 59% and 47% of cardiomyocytes within the BZ and infarct regions, respectively, at 14 days after MI (Figure 1A and 1B). With regard to other cardiac cell types, Runx1 expression significantly increased to 14% of noncardiomyocytes within the infarct region at 1 day after MI (Figure 1A and 1C) and increased further to 35% and 26% of noncardiomyocytes within the BZ and infarct regions, respectively, at 14 days after MI (Figure 1A and 1C). These data supported separate experiments that found an increased *Runx1* level at 1 and 14 days after MI in cardiomyocytes isolated from whole hearts and separated from other cell types as measured with real-time quantitative PCR data even though these data did not provide spatial resolution (Figure 1D).

### Direct Assessment of *Runx1* Function in Cardiomyocytes

To directly determine the contribution of Runx1 in cardiomyocytes to reduced cardiac function, we generated cardiomyocyte-specific *Runx1*-deficient mice using Cre-LoxP–based gene targeting strategies (online-only Data Supplement and Figure V in the online-only Data Supplement).^9^ Three groups of mice were generated: *Runx1*^*Δ/Δ*^ mice (*αMHC-MerCreMer:Runx1*^*fl/fl*^), littermate *Runx1*^*fl/fl*^ mice controlling for the insertion of the LoxP sites,^8^ and *Runx1*^*wt/wt*^ mice controlling for insertion of the tamoxifen-inducible *Cre* recombinase (α*MHC-MerCreMer:Runx1*^*wt/wt*^).^9^ Cardiomyocyte-specific excision of *Runx1* was induced in adult mice by a single intraperitoneal injection of tamoxifen (40 mg/kg). PCR of genomic DNA, real-time quantitative PCR, and Western blot analysis were performed on isolated cardiomyocytes and confirmed successful deletion of the *Runx1* gene after injection with tamoxifen (Figure V in the online-only Data Supplement).

### In Vivo Echocardiographic Assessment of *Runx1*^*Δ/Δ*^ Mice After MI

To establish whether LV systolic function was altered in *Runx1*^*Δ/Δ*^ mice after MI, we used M-mode echocardiography.

#### Cardiac Function

**Figure 2.**
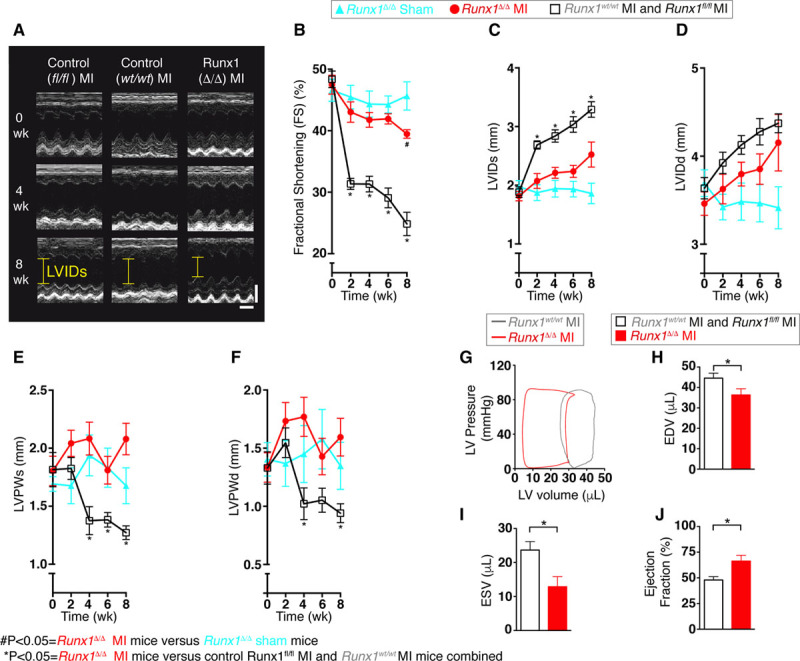
**Cardiac function in *Runx1*^*Δ/Δ*^ mice.** **A**, Echocardiography (scale: *x*=0.1 s; *y*=2 mm). **B**, The 8-week echocardiographic data for fractional shortening (FS) and **(C)** left ventricular (LV) internal diameter (LVID) at systole (LVIDs), **(D)** LVID at diastole (LVIDd), **(E)** LV posterior wall thickness at systole (LVPWs), and **(F)** LVPW thickness at diastole (LVPWd). (*Runx1*^*fl/fl*^ myocardial infarction [MI] and *Runx1*^*wt/wt*^ MI combined [n=11], *Runx1*^*Δ/Δ*^ MI [n=9], and *Runx1*^*Δ/Δ*^ sham [n=5]; ANOVA.) #*P*<0.05, *Runx1*^*Δ/Δ*^ MI mice vs. *Runx1*^*Δ/Δ*^ sham mice. *P<0.05, *Runx1*^*Δ/Δ*^ MI mice vs. control *Runx1*^*fl/fl*^ MI and *Runx1*^*wt/wt*^ MI mice combined. **G**, Pressure-volume (PV) loops of *Runx1*^*wt/wt*^ and *Runx1*^*Δ/Δ*^ 2 weeks after MI. **H**, Mean PV data 2 weeks after MI, end-diastolic volume (EDV), **(I)** end-systolic volume (ESV), and **(J)** ejection fraction (EF; *Runx1*^*fl/fl*^ MI and *Runx1*^*wt/wt*^ MI combined [n=11], *Runx1*^*Δ/Δ*^ MI [n=8]). *P<0.05, Student *t* test.

MI was surgically induced in *Runx1*^*Δ/Δ*^, *Runx1*^*fl/fl*^, and *Runx1*^*wt/wt*^ mice 1 week after tamoxifen injection in all mice. Echocardiography was performed before MI and every 2 weeks after MI to assess cardiac contractile function (Figure 2A). As expected, cardiac systolic function (assessed by fractional shortening) decreased in both groups of control mice (*Runx1*^*fl/fl*^ and *Runx1*^*wt/wt*^) after MI (Figure 2B). In contrast, *Runx1*^*Δ/Δ*^ mice demonstrated a markedly preserved fractional shortening that was 158% of the control mice at 8 weeks after MI (39.5±0.7% versus 24.9±1.9%; *P*<0.05; Figure 2A and 2B). *Runx1*^*Δ/Δ*^ mice undergoing a sham procedure 1 week after tamoxifen injection demonstrated no change in fractional shortening over the equivalent 8-week time course and were not significantly different from the *Runx1*^*Δ/Δ*^ MI mice until the 8-week time point (Figure 2B). The improved fractional shortening in *Runx1*^*Δ/Δ*^ mice after MI was the culmination of substantially improved cardiac contraction, as evidenced by the smaller LV internal diameter measured at systole (Figure 2A and 2C), which was 77% of the 2 control groups after MI (2.5±0.2 versus 3.3±0.1 mm; *P*<0.05). *Runx1*^*Δ/Δ*^ mice undergoing a sham procedure after tamoxifen administration demonstrated no change in LV internal diameter measured at systole over the equivalent 8-week time course. The LV internal diameter measured at diastole within the BZ region indicated that the hearts of both control and *Runx1*^*Δ/Δ*^ mice after MI dilated at this level of the myocardium, albeit to a lesser degree in *Runx1*^*Δ/Δ*^ mice (Figure 2D).

#### Cardiac Structure

As expected, LV posterior wall thickness during systole measured at the level of the BZ of control mice (*Runx1*^*fl/fl*^ and *Runx1*^*wt/wt*^) thinned after the 2-week post-MI time point as a result of the cardiac remodeling process (Figure 2E). In contrast, *Runx1*^*Δ/Δ*^ mice displayed preserved wall thickness that was 164% of control mice (*Runx1*^*fl/fl*^ and *Runx1*^*wt/wt*^) at 8 weeks after MI (2.07±0.14 versus 1.27±0.06 mm; *P*<0.05; Figure 2E). *Runx1*^*Δ/Δ*^ mice undergoing a sham procedure after MI demonstrated no change in LV posterior wall thickness during systole over the equivalent 8-week time course. The wall thickness data were confirmed at diastole 8 weeks after MI (Figure 2F).

These data were confirmed in a separate blinded study (Figure VIA–VIE in the online-only Data Supplement) in which the operator was blinded to the animals undergoing surgery, echocardiography, and analysis before MI and at 2 weeks after MI and at earlier time points at and before 1 week after MI (Figure VII in the online-only Data Supplement).

### In Vivo Ventricular Luminal Volumes and Ejection Fraction in *Runx1*^*Δ/Δ*^ Mice 2 Weeks After MI

LV ventricular luminal volume of the *Runx1*^*Δ/Δ*^ and control mice was assessed in vivo at 2 weeks after MI with PV loops (Figure 2G). The end-diastolic volume in the *Runx1*^*Δ/Δ*^ mice was reduced to 82% of that in control mice (*Runx1*^*fl/fl*^ and *Runx1*^*wt/wt*^), indicating a reduction in LV dilation (36.3±3.00 versus 44.5±2.47 μL; *P*<0.05; Figure 2G and 2H). The end-systolic volume in *Runx1*^*Δ/Δ*^ mice was reduced to 54% of the control mice, indicating a greater level of emptying of LV blood volume (12.9±2.98 versus 23.7±2.51 μL; *P*<0.05; Figure 2G and 2I). This leftward shift in the PV loop in the *Runx1*^*Δ/Δ*^ mice resulted in an ejection fraction that was 138% of the control mice (66.3±5.69% versus 48.0±3.18%; *P*<0.05; Figure 2G and 2J).

### Histological Assessment of *Runx1*^*Δ/Δ*^ Mice 8 Weeks After MI

#### Heart Structure

**Figure 3.**
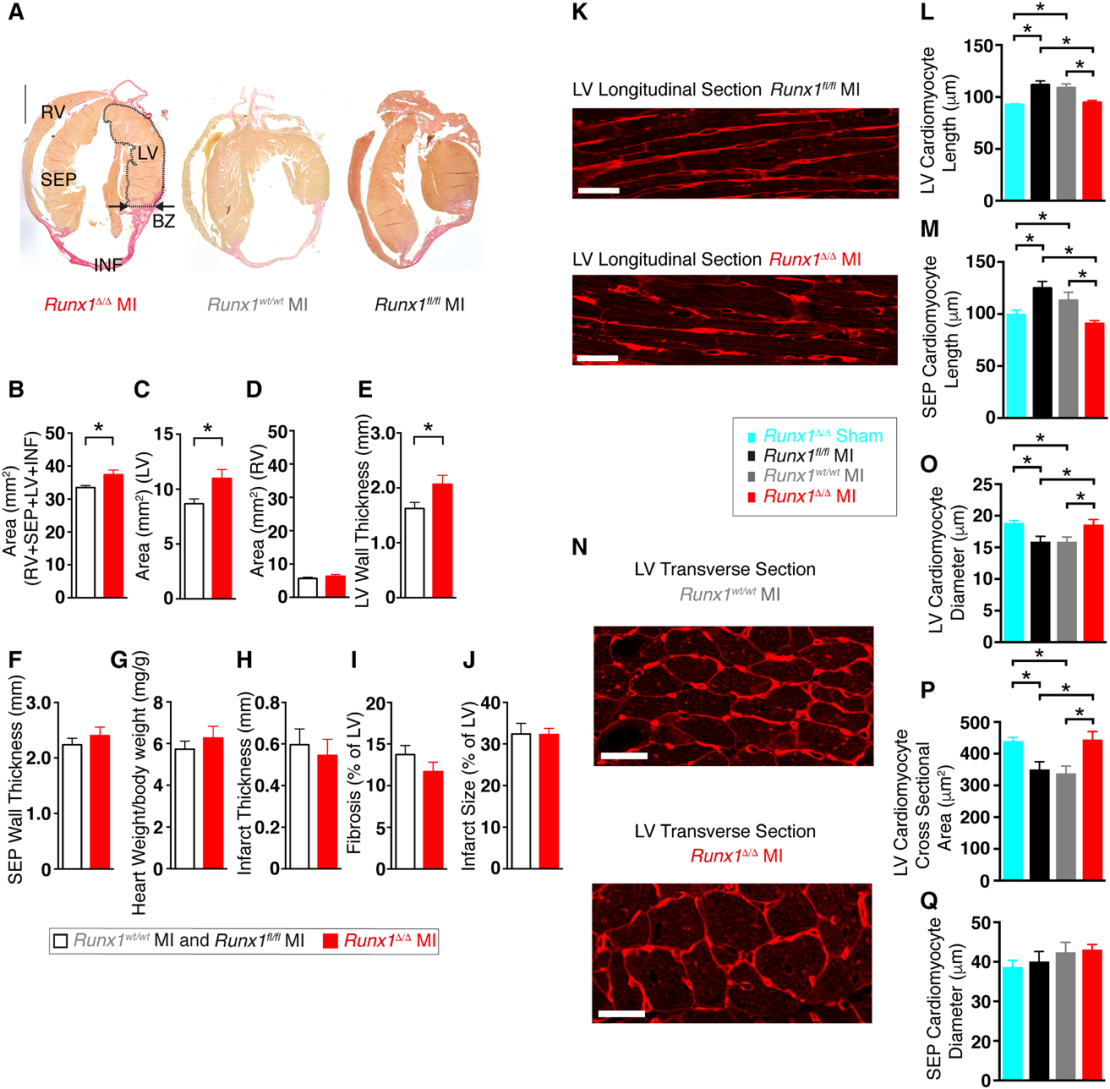
***Runx1*^*Δ/Δ*^ mice cardiac structure 8 weeks after myocardial infarction (MI).** **A**, Picrosirius Red–stained hearts. Scale, 1 mm. BZ indicates border zone; INF, infarct; LV, left ventricle; RV, right ventricle; and SEP, septum. Mean **(B)** area of whole heart (all regions), **(C)** LV, **(D)** RV, and **(E)** LV wall thickness at BZ region; **(F)** septum wall thickness at BZ region level; **(G)** ratio of heart weight to body weight; **(H)** infarct thickness; **(I)** LV fibrosis; and **(J)** infarct size (*Runx1*^*fl/fl*^ MI and *Runx1*^*wt/wt*^ MI combined [n=12], *Runx1*^*Δ/Δ*^ MI [n=5]). *P<0.05, Student *t* test. **K**, Wheat germ agglutinin (WGA) staining of LV cardiomyocytes (longitudinal) of *Runx1*^*fl/fl*^ MI (top) and *Runx1*^*Δ/Δ*^ (bottom) after MI (scale bar, 25 mm). **L**, Mean LV cardiomyocyte length (*Runx1*^*Δ/Δ*^ sham [n=55 cardiomyocytes, n=3 hearts], *Runx1*^*fl/fl*^ MI [n=122 cardiomyocytes, n=6 hearts], *Runx1*^*wt/wt*^ MI [n=109 cardiomyocytes, n=6 hearts], *Runx1*^*Δ/Δ*^ MI [n=102 cardiomyocytes, n=6 hearts]). *P<0.05, linear mixed modeling. **M**, Mean SEP cardiomyocyte length (*Runx1*^*Δ/Δ*^ sham [n=29 cardiomyocytes, n=3 hearts], *Runx1*^*fl/fl*^ MI [n=82 cardiomyocytes, n=6 hearts], *Runx1*^*wt/wt*^ MI [n=84 cardiomyocytes, n=6 hearts], *Runx1*^*Δ/Δ*^ MI [n=64 cardiomyocytes, n=6 hearts]). **N**, WGA staining of LV cardiomyocytes (transverse) of *Runx1*^*wt/wt*^ MI (left) and *Runx1*^*Δ/Δ*^ MI (right; scale bar, 25 mm). **O**, Mean LV cardiomyocyte diameter (*Runx1*^*Δ/Δ*^ sham [n=449 cardiomyocytes, n=3 hearts], *Runx1*^*fl/fl*^ MI [n=811 cardiomyocytes, n=6 hearts], *Runx1*^*wt/wt*^ MI [n=897 cardiomyocytes, n=6 hearts], *Runx1*^*Δ/Δ*^ MI [n=878 cardiomyocytes, n=6 hearts]). **P**, Mean LV cardiomyocyte cross-sectional area (*Runx1*^*Δ/Δ*^ sham [n=403 cardiomyocytes, n=3 hearts], *Runx1*^*fl/fl*^ MI [n=714 cardiomyocytes, n=6 hearts], *Runx1*^*wt/wt*^ MI [n=785 cardiomyocytes, n=6 hearts], *Runx1*^*Δ/Δ*^ MI [n=699 cardiomyocytes, n=6 hearts]). **Q**, Mean SEP cardiomyocyte diameter (*Runx1*^*Δ/Δ*^ sham [n=238 cardiomyocytes, n=3 hearts], *Runx1*^*fl/fl*^ MI [n=465 cardiomyocytes, n=6 hearts], *Runx1*^*wt/wt*^ MI [n=454 cardiomyocytes, n=6 hearts], *Runx1*^*Δ/Δ*^ MI [n=452 cardiomyocytes, n=6 hearts]). *P<0.05, linear mixed modeling.

We next investigated whether an altered whole-heart structure contributed to the preserved cardiac performance of *Runx1*^*Δ/Δ*^ mice after MI. Analysis of the different regions of the heart (Figure 3A) with Picrosirius Red staining of serial sections of hearts from *Runx1*^*Δ/Δ*^ mice 8 weeks after MI showed that the mean 2-dimensional whole-heart area (RV+septum+LV+infarct) was 112% of control mice (*Runx1*^*fl/fl*^ and *Runx1*^*wt/wt*^) after MI (37.4±1.4 versus 33.5±0.6 mm^2^; *P*<0.05; Figure 3B). This increase in heart area was associated with the LV free wall (Figure 3A, dotted area), which in *Runx1*^*Δ/Δ*^ mice after MI was 127% of control mice after MI (11.0±0.8 versus 8.7±0.4 mm^2^; *P*<0.05; Figure 3C). No change was detected in the RV wall area in *Runx1*^*Δ/Δ*^ mice after MI (Figure 3D); therefore, further investigation focused on the structure of the LV.

LV wall thickness (measured at the level of the BZ; Figure 3A, arrows) in *Runx1*^*Δ/Δ*^ mice after MI was 127% of control mice after MI (2.07±0.2 versus 1.63±0.1 mm; *P*<0.05; Figure 3E), a finding that supported the echocardiographic data (Figure 2E). No change was detected in the septal wall thickness in *Runx1*^*Δ/Δ*^ mice after MI (Figure 3F) or overall heart weight (Figure 3G). Infarct thickness and fibrosis (Figure 3H and 3I) were not different in *Runx1*^*Δ/Δ*^ mice after MI versus control mice after MI. Furthermore, infarct size (32.3±1.5% versus 32.7±3.1% versus 31.9±5% of LV; Runx1^Δ/Δ^ [N=5] versus Runx1^wt/wt^ [N=7] versus Runx1^fl/fl^ [N=5]; *P*>0.05; Figure 3J) at 8 weeks after MI (and the earlier time point of 24 hours after MI; Figure VIIIA and VIIIB in the online-only Data Supplement) was not different in *Runx1*^*Δ/Δ*^ versus control mice and therefore did not explain the preserved LV function observed in vivo (Figure 2B).

#### Cardiomyocyte Size

To investigate why LV free wall thickness was preserved in *Runx1*^*Δ/Δ*^ mice 8 weeks after MI relative to the wall thinning observed in control mice after MI (Figure 2E and 2F), the cardiomyocyte size in *Runx1*^*Δ/Δ*^ mice 8 weeks after MI was determined with wheat germ agglutinin staining. As expected, LV cardiomyocytes from control *Runx1*^*fl/fl*^ and *Runx1*^*wt/wt*^ mice 8 weeks after MI exhibited significant cell lengthening to 121% and 118% of *Runx1*^*Δ/Δ*^ sham mice (111.7±3.8 versus 108.8±3.7 versus 92.53±1.04 μm; *P*<0.05; Figure 3K and 3L). However, cardiomyocyte elongation was absent in *Runx1*^*Δ/Δ*^ mice at 8 weeks after MI (Figure 3K and 3L). An equivalent absence of cardiomyocyte lengthening was also observed in septal cardiomyocytes (Figure 3M). LV cardiomyocytes from control *Runx1*^*fl/fl*^ and *Runx1*^*wt/wt*^ mice 8 weeks after MI exhibited a significant decrease in cell diameter to 86% and 85% of *Runx1*^*Δ/Δ*^ sham mice (15.91±0.83 versus 15.88±0.75 versus 18.86±0.37 μm; *P*<0.05; Figure 3N and 3O). However, cardiomyocyte cell diameter did not decrease in *Runx1*^*Δ/Δ*^ mice 8 weeks after MI (Figure 3O). LV cardiomyocytes from control *Runx1*^*fl/fl*^ and *Runx1*^*wt/wt*^ mice 8 weeks after MI exhibited a significant decrease in cardiomyocyte cross-sectional area to 79% and 76% of *Runx1*^*Δ/Δ*^ sham mice (350.6±23.9 versus 338.2±22.6 versus 440.0±11.5 μm; *P*<0.05; Figure 3N and 3P). However, cardiomyocyte cell cross-sectional area did not decrease in *Runx1*^*Δ/Δ*^ mice 8 weeks after MI (Figure 3P). No change in septal cardiomyocyte diameter was observed at 8 weeks after MI in any group (Figure 3Q).

### Calcium Transients in *Runx1*^*Δ/Δ*^ Mice 2 Weeks After MI

Although increased fractional shortening paralleled increased wall thickness in *Runx1*^*Δ/Δ*^ mice at 8 weeks after MI (Figure 2B and 2E), we noted that wall thickness was not significantly different between the 3 groups at 2 weeks after MI. However, *Runx1*^*Δ/Δ*^ mice still exhibited greater fractional shortening at this time point than was observed for the 2 control groups. To investigate this dichotomy, we isolated cardiomyocytes from hearts at 2 weeks after MI to characterize calcium handling. This was achieved by measuring the [Ca^2+^]_i_, focusing on electrically induced SR-mediated calcium release (calcium transients) into the cytosol, which predominately determines the force of contraction.

**Figure 4.**
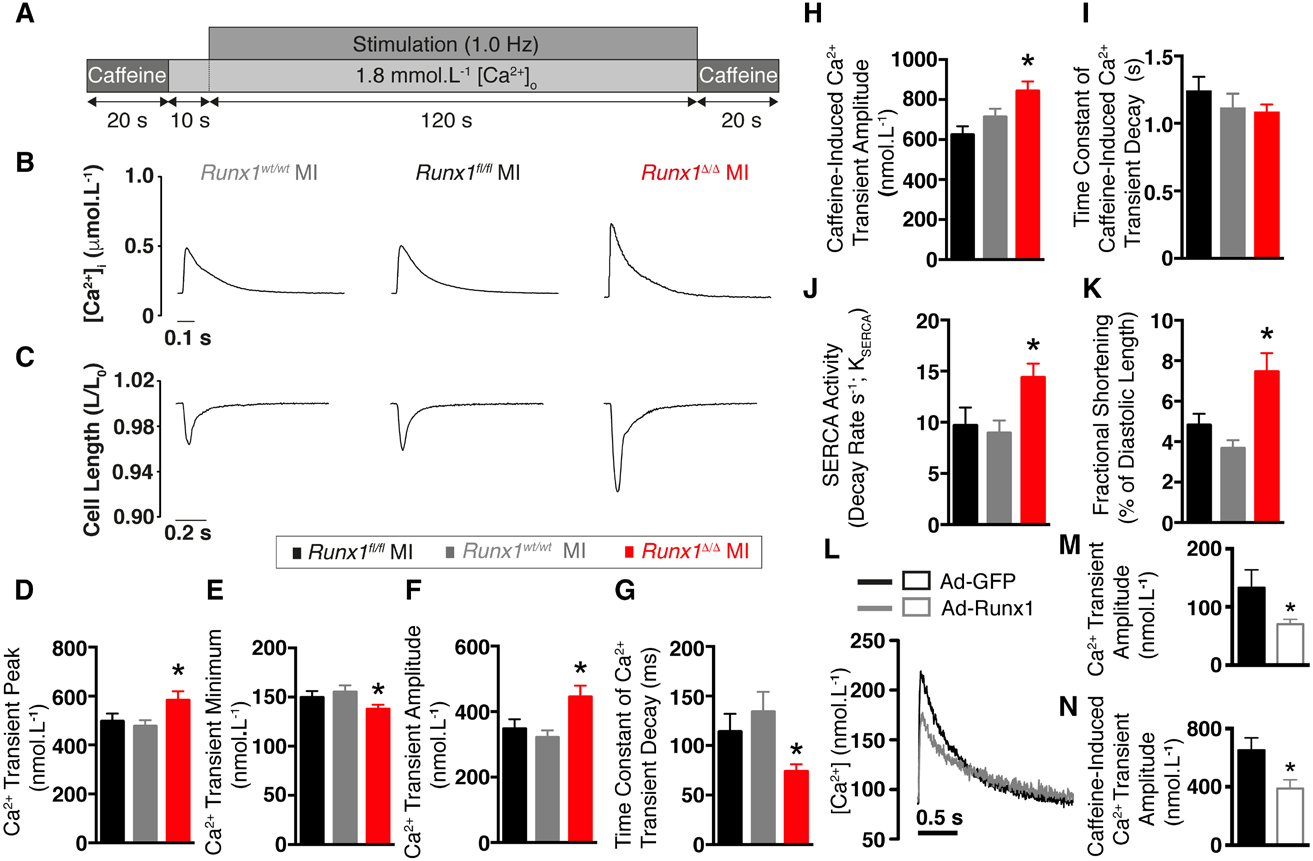
**Excitation-contraction coupling in *Runx1*^*Δ/Δ*^ mice 2 weeks after myocardial infarction (MI).** **A**, Protocol, **(B)** typical calcium (Ca^2+^) transients, and **(C)** cell shortening. Mean Ca^2+^ transient: **(D)** peak, **(E)** minimum, and **(F)** amplitude (*Runx1*^*fl/fl*^ MI [n=25 cardiomyocytes, n=3 hearts], *Runx1*^*wt/wt*^ MI [n=28 cardiomyocytes, n=4 hearts], *Runx1*^*Δ/Δ*^ MI [n=28 cardiomyocytes, n=3 hearts]). *P<0.05, *Runx1*^*Δ/Δ*^ MI vs. *Runx1*^*fl/fl*^ MI and *Runx1*^*wt/wt*^ MI combined, linear mixed modeling. **G**, Mean time constant of Ca^2+^ transient decay. **H**, Mean caffeine-induced Ca^2+^ transient amplitude. **I**, Mean time constant of decay for caffeine-induced Ca^2+^ transient amplitude. **J**, Mean sarcoendoplasmic reticulum calcium transport ATPase (SERCA) activity. **K**, Mean fractional shortening (*Runx1*^*fl/fl*^ MI [n=27 cardiomyocytes, n=3 hearts], *Runx1*^*wt/wt*^ MI [n=27 cardiomyocytes, n=4 hearts], *Runx1*^*Δ/Δ*^ MI [n=23 cardiomyocytes, n=3 hearts]). **L**, Ca^2+^ transients from cardiomyocytes transduced with enhanced green fluorescent protein (Ad-GFP) or Ad-Runx1. **M**, Mean Ca^2+^ transient peak; Ad-GFP (n=16 cardiomyocytes, n=6 hearts) and Ad-Runx1 (n=22 cardiomyocytes, n=6 hearts). *P<0.05, linear mixed modeling. **N**, Caffeine-induced Ca^2+^ transient amplitude, Ad-GFP (n=13 cardiomyocytes, n=6 hearts), and Ad-Runx1 (n=19 cardiomyocytes, n=6 hearts). *P<0.05, linear mixed modeling.

Cardiomyocytes isolated at 2 weeks after MI were stimulated at 1.0 Hz to elicit calcium transients and cell shortening (Figure 4A–4C). The calcium transient peak (systolic [Ca^2+^]_i_) in *Runx1*^*Δ/Δ*^ mice was 117% and 122% of control (*Runx1*^*fl/fl*^ and *Runx1*^*wt/wt*^) mice (582.8±36.1 versus 497.4±31.3 versus 477.0±24.5 nmol/L [Ca^2+^]_i_; *P*<0.05; Figure 4B and 4D). The calcium transient minimum (diastolic [Ca^2+^]_i_) in *Runx1*^*Δ/Δ*^ mice was 92% and 89% of control (*Runx1*^*fl/fl*^ and *Runx1*^*wt/wt*^) mice (137.8±4.5 versus 149.8±6.3 versus 155.1±6.8 nmol/L [Ca^2+^]_i_; *P*<0.05; Figure 4B and 4E). The changes in peak and minimum [Ca^2+^]_i_ of *Runx1*^*Δ/Δ*^ mice resulted in a calcium transient amplitude that was 128% and 138% of control (*Runx1*^*fl/fl*^ and *Runx1*^*wt/wt*^) mice (445.0±34.3 versus 347.5±29.0 versus 321.9±20.7nmol/L [Ca^2+^]_i_; *P*<0.05; Figure 4B and 4F). Furthermore, the time constant of calcium transient decay in the *Runx1*^*Δ/Δ*^ mice was 65% and 55% of control (*Runx1*^*fl/fl*^ and *Runx1*^*wt/wt*^) mice (0.074±0.007 versus 0.114±0.018 versus 0.134±0.020 s; *P*<0.05; Figure 4B and 4G), suggesting an increased rate of removal of calcium from the cytosol.

The increased calcium transient amplitude in *Runx1*^*Δ/Δ*^ mice after MI occurred in the absence of any significant changes in calcium entry or action potential duration (as measured indirectly with the QT interval on the ECG or directly with voltage measurements on isolated cardiomyocytes at 2 weeks after MI; Figure IX in the online-only Data Supplement).

#### Caffeine-Induced Calcium Transients and Cell Shortening

We hypothesized that the lowered time constant of decay detected in the *Runx1*^*Δ/Δ*^ mice 2 weeks after MI might reflect either increased SR calcium uptake via sarco/endoplasmic reticulum Ca^2+^-ATPase (SERCA) or extrusion from the cell via the sodium-calcium exchanger. To address this issue, we applied a rapid bolus of 10 mmol/L caffeine at the end of the protocol to release all of the calcium from the SR into the cytosol. This approach enabled assessment of SR calcium content.

The SR calcium content of the *Runx1*^*Δ/Δ*^ mice was 135% and 118% of the control *Runx1*^*fl/fl*^ and *Runx1*^*wt/wt*^ mice, respectively (842.8±47.2 versus 624.3±42.2 versus 712.6±40.0 nmol/L [Ca^2+^]_i_; *P*<0.05; Figure 4H). SERCA-mediated calcium uptake is bypassed during application of 10 mmol/L caffeine, and cytosolic calcium removal occurs predominately via the sodium-calcium exchanger. The activity of the sodium-calcium exchanger, as assessed by the time constant of caffeine-induced calcium transient decay, was not different between the 3 groups (Figure 4I).

The increased SR calcium content observed in *Runx1*^*Δ/Δ*^ mice might reflect enhanced SERCA activity (K_SERCA_). Therefore, we measured the rate constant of decay of the caffeine-induced calcium transient (which includes sarcolemmal efflux but not SR calcium uptake) and subtracted this value from that of the electrically stimulated calcium transient (which includes both SR calcium uptake and sarcolemmal efflux).^14,15^ The K_SERCA_ of the *Runx1*^*Δ/Δ*^ mice was 148% and 160% of control *Runx1*^*fl/fl*^ and *Runx1*^*wt/wt*^ mice (14.4±1.4 versus 9.7±1.8 versus 9.0±1.2 s^−1^; *P*<0.05; Figure 4J). To corroborate that the increased calcium transient amplitude of *Runx1*^*Δ/Δ*^ mice resulted in increased cell shortening, we performed edge-detection shortening measurements (Figure 4C). Cardiomyocyte shortening in *Runx1*^*Δ/Δ*^ mice 2 weeks after MI was 156% and 203% of the control *Runx1*^*fl/fl*^ and *Runx1*^*wt/wt*^ mice (7.5±0.9% versus 4.8±0.6% versus 3.7±0.4% of diastolic length; *P*<0.05; Figure 4C and 4K).

#### Effect of Overexpressing Runx1 on Calcium Transient Amplitude and SR Calcium Content in Normal Cardiomyocytes

To further support the novel link between Runx1 and calcium handling in isolated cardiomyocytes, we performed a gain-of-function study by overexpressing Runx1 via adenoviral-mediated gene transfer (Ad-Runx1) in isolated adult cardiomyocytes from normal hearts. The calcium transient amplitude in Ad-Runx1–transduced cardiomyocytes was 53% of cardiomyocytes transduced with the control adenoviral vector expressing green fluorescent protein (70.5±8.3 versus 133.4±30.7 nmol/L [Ca^2+^]_i_; *P*<0.05; Figure 4L and 4M). SR calcium content in cardiomyocytes overexpressing Runx1 was 60% of the control cardiomyocytes (388.8±60.3 versus 651.8±84.4 nmol/L [Ca^2+^]_i_; *P*<0.05; Figure 4N).

### Expression of Calcium Handling Proteins in *Runx1*^*Δ/Δ*^ Mice 2 Weeks After MI

To investigate the mechanism by which SERCA activity is increased, we quantified the expression and phosphorylation levels of key calcium handling proteins involved in the control of SERCA-mediated calcium uptake in isolated cardiomyocytes 2 weeks after MI.

**Figure 5.**
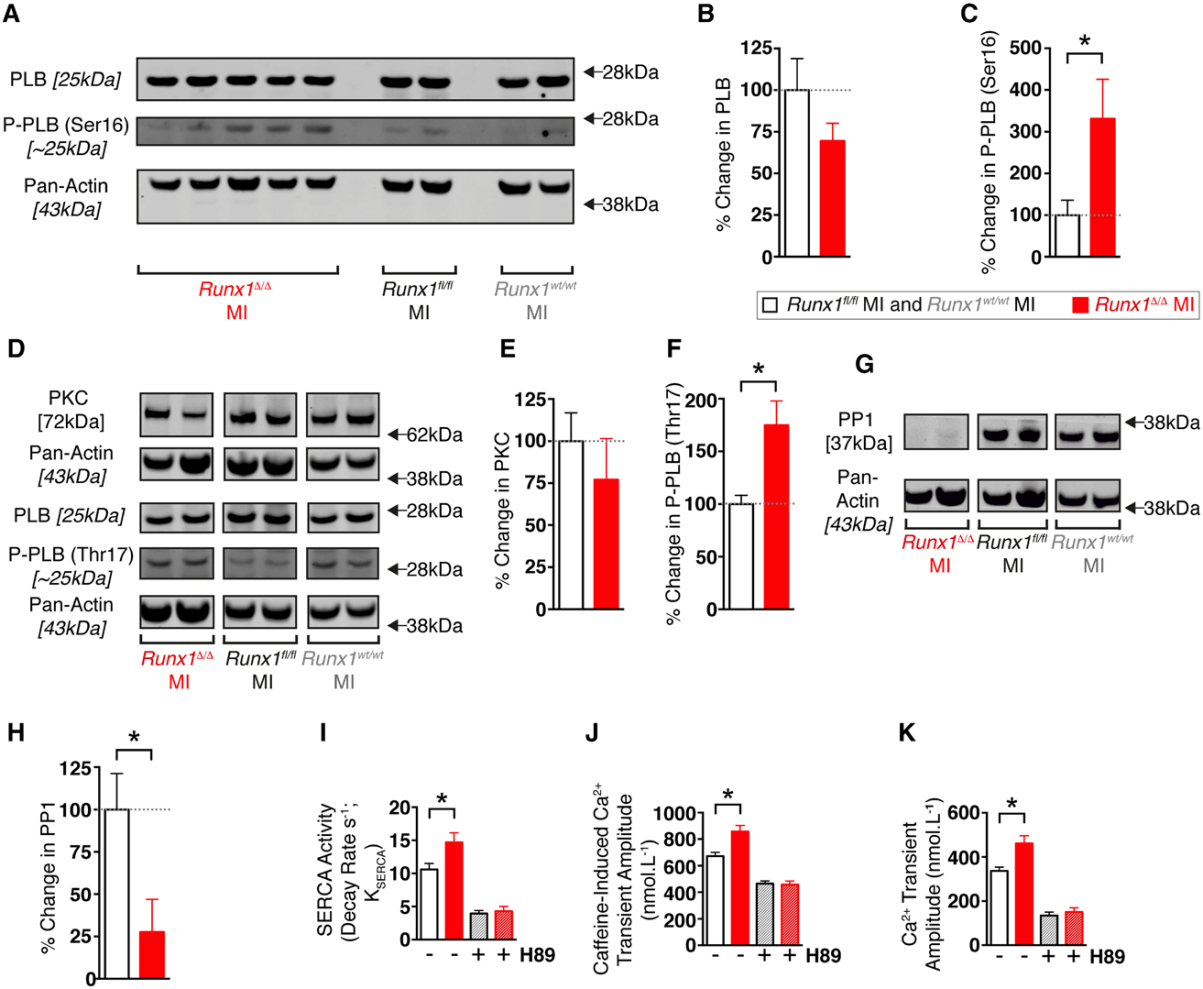
**Phospholamban (PLB) regulation in isolated cardiomyocytes from *Runx1*^*Δ/Δ*^ mice 2 weeks after myocardial infarction (MI).** **A**, Western blot of PLB, phosphorylation of PLB at serine-16 [P-PLB (Ser16)], and pan-actin loading control. Percentage change in protein for **(B)** PLB and **(C)** P-PLB (Ser16/total PLB; *Runx1*^*Δ/Δ*^ MI [n=5 hearts] vs. *Runx1*^*wt/wt*^ and *Runx1*^*fl/fl*^ MI [n=5 hearts]). *P<0.05, Student *t* test. **D**, Western blot of protein kinase C (PKC), PLB, and PLB phosphorylation at threonine-17 [P-PLB (Thr17)]. Percentage change in protein for **(E)** PKC and **(F)** P-PLB (Thr17/total PLB; *Runx1*^*Δ/Δ*^ MI [n=5 hearts] vs. *Runx1*^*wt/wt*^ MI and *Runx1*^*fl/fl*^ MI [n=9 hearts]). *P<0.05, Student *t* test. **G**, Western blot of protein phosphatase 1 (PP1). **H**, Percentage change in PP1 (*Runx1*^*Δ/Δ*^ MI [n=5 hearts] vs. *Runx1*^*wt/wt*^ MI and *Runx1*^*fl/fl*^ MI [n=9 hearts]). **I** through **K**, Mean sarcoendoplasmic reticulum calcium transport ATPase (SERCA) activity, caffeine-induced calcium transient amplitude, and calcium transient amplitude data from Figure 4 compared with mean data obtained from 2-week post-MI isolated cardiomyocytes with H89 (hatched white column, *Runx1*^*wt/wt*^ MI and *Runx1*^*fl/fl*^ MI+H89 [n=16 cardiomyocytes, 3 hearts]; hatched red column, *Runx1*^*Δ/Δ*^ MI+H89 [n=15 cardiomyocytes, 3 hearts]). *P<0.05, Student *t* test.

Levels of phospholamban, an inhibitory protein that regulates SERCA activity, were not significantly altered in the *Runx1*^*Δ/Δ*^ mice (Figure 5A and 5B). In contrast, phosphorylation of phospholamban (which relieves SERCA inhibition and improves cardiac contractility) at the PKA-target residue Ser16 was 331% of control mice (*Runx1*^*wt/wt*^ and *Runx1*^*fl/fl*^; 331.2±94.5% versus 100±35.6% change; *P*<0.05; Figure 5A and 5C).

Decreased levels of protein kinase C indirectly enable enhanced phosphorylation of phospholamban and increase cardiac contractility^16^; however, no between-group differences were detected in the levels of protein kinase C (Figure 5D and 5E). Phosphorylation of phospholamban at the Ca^2+^/calmodulin-dependent protein kinase II target residue threonine-17 in *Runx1*^*Δ/Δ*^ mice was 175% of control mice (*Runx1*^*wt/wt*^ and *Runx1*^*fl/fl*^; 175.0±22.9% versus 100±8.2% change; *P*<0.05; Figure 5D and 5F). A possible regulator of phosphorylation of phospholamban is protein phosphatase 1, which dephosphorylates phospholamban.^16^ We found that expression of protein phosphatase 1 in *Runx1*^*Δ/Δ*^ mice was decreased to 28% of control mice (*Runx1*^*wt/wt*^ and *Runx1*^*fl/fl*^) after MI (27.6±19.4% versus 100±21.3% change; *P*<0.05; Figure 5G and 5H).

To confirm that the increased SERCA activity, SR calcium content, and calcium transient amplitude observed in *Runx1*^*Δ/Δ*^ mice 2 weeks after MI (Figure 4) were PKA-mediated, we investigated the effect of the PKA inhibitor (H89) on calcium handling. The addition of H89 completely blocked enhancement of all 3 parameters in the *Runx1*^*Δ/Δ*^ mice relative to the control *Runx1*^*wt/wt*^ and *Runx1*^*fl/fl*^ mice (Figure 5I–5K).

### Cardiac Contractility in *Runx1*^*Δ/Δ*^ Mice After Ischemia With Reperfusion

Reperfusion of a blocked coronary artery limits cell death after MI; this effect can be achieved clinically via percutaneous coronary intervention. We therefore tested whether *Runx1*^*Δ/Δ*^ mice also maintain a preserved LV contractile function in an additional clinically relevant model of ischemia with reperfusion.

**Figure 6.**
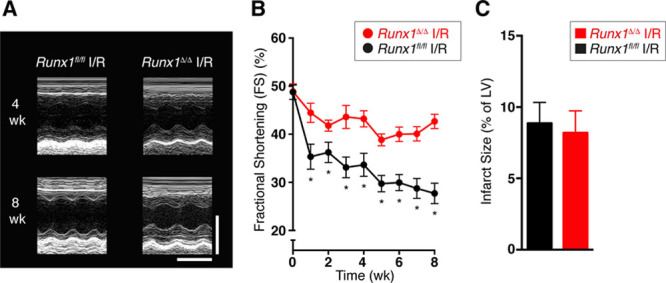
***Runx1*^*Δ/Δ*^ mice after ischemia with reperfusion (I/R).** **A**, Echocardiographic images (scale: *x*=0.1 s; *y*=2 mm). **B**, Mean echocardiographic fractional shortening (FS) data of *Runx1*^*fl/fl*^ I/R (n=9) and *Runx1*^*Δ/Δ*^ I/R (n=8). *P<0.05, Student *t* test. **C**, Mean infarct size (*Runx1*^*fl/fl*^ I/R [n=9] and *Runx1*^*Δ/Δ*^ I/R [n=7]). Student *t* test. LV indicates left ventricular.

The left anterior descending coronary artery was temporarily ligated in vivo for 45 minutes followed by reperfusion, and the *Runx1*^*Δ/Δ*^ mice recovered for 8 weeks. Fractional shortening was assessed with echocardiography before MI and weekly after the induction of MI with reperfusion. As expected, fractional shortening decreased in control *Runx1*^*fl/fl*^ mice after reperfusion (Figure 6A and 6B). In contrast, *Runx1*^*Δ/Δ*^ mice demonstrated markedly preserved fractional shortening, which was 154% of control *Runx1*^*fl/fl*^ mice at 8 weeks after reperfusion (42.7±1.5% versus 27.7±2.13%; *P*<0.05; Figure 6A and 6B). As with the animal model of permanent coronary artery ligation, infarct size at 8 weeks after reperfusion (and the earlier time point of 24 hours after reperfusion) was not different in *Runx1*^*Δ/Δ*^ versus control mice (Figure 6C and Figure VIIIC and VIIID in the online-only Data Supplement).

## Discussion

Runx1 has been most intensively studied in the hematopoietic system because its function is frequently corrupted in different subtypes of leukemia. Although it is known to have a role in lineage differentiation and tissue function in a range of other systems, there is almost no information relating to its role in adult cardiomyocytes other than the observation that it can be reactivated after myocardial insult.^5,7^ Our novel study addressed a vital question: Is increased expression of RUNX1 after MI merely a marker of ischemic damage, or does it play a functional role in adult cardiomyocytes after MI? We provide new evidence that *Runx1* has an important role in cardiomyocytes after MI. Reducing *Runx1* function preserved cardiac contractility and prevented adverse cardiac remodeling, which suggests that targeting the actions of this gene could have important implications for patient survival after MI. This research transcends discipline boundaries because it not only widens the importance of Runx1 to other fields of medicine but also describes a novel function for this gene.

Our results provide the first detailed quantification of regional *Runx1* expression in mouse heart tissue after MI. At 4 weeks after MI, *Runx1* mRNA was increased within the BZ myocardium and infarct region, which was sustained until at least 8 weeks after MI, at which time *Runx1* expression also increased within the remote LV myocardium. This is important given that changes in Runx1 expression at the mRNA and protein levels are not restricted to rodent MI models but also occur in patients with MI.^5,7^ In separate experiments, we were able to demonstrate that *Runx1* expression is increased within the BZ and infarct region after MI within the contractile elements of the heart—that is, the cardiomyocytes—as early as 1 to 14 days after MI (Figure 1).

To determine the specific contribution of Runx1 in cardiomyocytes to reduced cardiac contractility, we generated a new tamoxifen-inducible cardiomyocyte-specific Runx1-deficient mouse with the hypothesis that these mice would demonstrate improved cardiac function. Induction of MI in control transgenic mice led to the expected LV wall thinning, cardiac dilation, and reduced contractility 8 weeks after MI. However, all of these adverse cardiac remodeling parameters were absent or reduced in *Runx1*^*Δ/Δ*^ mice at this time point. One possible explanation for the observed preservation of systolic function could have been a reduction in the infarct size of *Runx1*^*Δ/Δ*^ mice given that infarct size correlates with systolic function.^17^ However, *Runx1*^*Δ/Δ*^ mice exhibited preservation of geometric shape and contractility after MI, with no difference in infarct size (at both early and late time points) or fibrosis compared with control mice.

To establish the mechanism underlying these notable findings, we first investigated cardiomyocyte size. Control mice demonstrated the expected cardiomyocyte lengthening and thinning (eccentric hypertrophy) at 8 weeks after MI.^1^ However, these changes were absent in *Runx1*^*Δ/Δ*^ mice. Such protection against eccentric hypertrophy at 8 weeks after MI seems highly likely to have afforded *Runx1*^*Δ/Δ*^ mice protection from ventricular dilation and thinning, ultimately leading to preserved contractility. Nevertheless, at 2 weeks after MI, the wall thickness in *Runx1*^*Δ/Δ*^ mice was comparable to that of control mice (because wall thinning in control mice had not yet begun), but contractile function was still dramatically improved. This finding indicated that prevention of wall thinning and dilation could not fully explain the preserved contractile function observed at 2 weeks after MI.

The above dichotomy led us to investigate calcium handling in cardiomyocytes isolated from *Runx1*^*Δ/Δ*^ mice 2 weeks after MI, in particular electrically stimulated calcium release (the calcium transient) from the intracardiomyocyte calcium store (the SR) and subsequent cell shortening. Patients and animal models with MI typically exhibit calcium transients with lower amplitude and a slower rate of decline than control/healthy cardiomyocytes, an observation largely attributed to reduced SR-mediated calcium uptake via SERCA.^15^ *Runx1*^*Δ/Δ*^ mice exhibited increased calcium transient amplitude and reduced time constant of decline after MI compared with control mice after MI, resulting in an increase in cell shortening. The accompanying higher SR calcium content observed in *Runx1*^*Δ/Δ*^ mice after MI can explain the enhanced calcium transient amplitude^18^ because equalizing the SR calcium content with H89 resulted in a calcium transient equivalent to that of control.

Analysis of the caffeine-induced calcium transient found no detectable change in sodium-calcium exchanger activity in *Runx1*^*Δ/Δ*^ mice after MI compared with control mice after MI. However, enhanced SR-mediated calcium uptake via SERCA was observed in the *Runx1*^*Δ/Δ*^ mice. SERCA activity is a major determinant of the SR calcium content. Furthermore, this pump is regulated predominately by the inhibitory protein phospholamban. Although expression of phospholamban was not altered in *Runx1*^*Δ/Δ*^ mice after MI, we explored some of the proteins that regulate phospholamban activity.^16^

Phospholamban-mediated inhibition of SERCA is balanced by phosphorylation by PKA and Ca^2+^/calmodulin-dependent protein kinase II (which relieves SERCA inhibition) and dephosphorylation by protein phosphatase 1 (which returns phospholamban to its inhibitory state^16^). We found that ventricular cardiomyocytes from *Runx1*^*Δ/Δ*^ mice exhibited increased PKA-mediated phosphorylation of phospholamban, possibly as a result of reduced levels of protein phosphatase 1. These mechanistic data suggest that phospholamban phosphorylation stimulates SERCA activity in *Runx1*^*Δ/Δ*^ mice after MI and leads to an increased SR calcium content, which in turn increases electrically induced SR-mediated calcium release and doubles cardiomyocyte contraction. Our proposed mechanism was supported by complete blockage of the enhanced calcium transient in cardiomyocytes from *Runx1*^*Δ/Δ*^ mice 2 weeks after MI by inhibition of PKA. The enhanced rate of removal of calcium from the cytosol after increased SERCA activity is sufficient to reduce the end-diastolic [Ca^2+^]_i_, which not only improves whole-heart relaxation but also may limit the stimulation of hypertrophic factors.^1^

Previous studies strongly support our proposed mechanism that the effect of Runx1 on SR function is a major contributor to the beneficial effects observed in *Runx1*^*Δ/Δ*^ mice after MI. Decreased SR function has been demonstrated among patients with heart failure,^15^ and enhanced SR-mediated calcium cycling markedly preserved contractility, reduced adverse cardiac remodeling, and delayed progression to heart failure at levels not dissimilar from those observed in the present study.^16,19^

The key findings of our study will likely initiate further research into the beneficial effects of decreasing Runx1 expression in alternative animal models of cardiac disease. As a testament to this goal, we found that *Runx1*^*Δ/Δ*^ mice are protected from adverse cardiac remodeling in a separate clinically relevant surgical model. In this model, the blocked coronary artery was subsequently unblocked after a period of ischemia, as would be the case for patients undergoing percutaneous coronary intervention. These additional data further support our study and the translational potential of this new target.

Although our strategy did not result in inactivation of the *Runx1* gene in all cardiomyocytes, we postulate that cardiac function improves when only a subset of cardiomyocytes benefit from better Ca^2+^ handling after inactivation of Runx1. Because it is more feasible in a clinical setting to suppress genes in a subset of cardiomyocytes rather than in all cells, we think Runx1 or targets showing a similar potency are particularly attractive for therapeutic interventions.

### Conclusions

We have demonstrated for the first time that Runx1 modulates cardiac SR calcium uptake and contractile function. Reducing Runx1 function drives increased contractility after MI, thereby preserving LV systolic function and preventing adverse cardiac remodeling. Clinical studies clearly demonstrate that preserving cardiac contractility and protecting against adverse cardiac remodeling are key factors in limiting the progression from MI to heart failure.^2^ Identification of a new therapeutic target that achieves this objective is urgently required. To this end, we envisage that Runx1 will be exploited in future basic and translational studies to limit the progression of patients with MI to heart failure, thereby improving survival rates and quality of life.

## Acknowledgments

The authors thank L. de Windt (Maastricht University, Netherlands) for the gift of *αMHC-MerCreMer*:*Runx1*^*wt/wt*^ mice; N. Speck (University of Pennsylvania) for the gift of *Runx1*^*fl/fl*^ mice; S. Stifani (McGill University, Canada) for the gift of Ad-Runx1; J. Neil (University of Glasgow, UK) for contributions to the manuscript; V. Heath for editorial assistance; J. McClure (University of Glasgow) for statistical advice; and A. Jenkins (University of Glasgow, UK), N. Mackay (University of Glasgow, UK), and M. Hughes (Beatson Institute, UK) for technical advice.

## Sources of Funding

Funding was received from the UK Medical Research Council (MR/M021459/1, MR/K501335/1), British Heart Foundation (PG/09/004), Cancer Research UK (C596/A17196), and University of Glasgow, UK.

## Disclosures

None.

## Supplementary Material
