# Supplementary material for: Runx1 Deficiency Protects Against Adverse Cardiac Remodeling After Myocardial Infarction
Source: Circulation. 2017 Dec 26;137(1):57–70. doi: 10.1161/CIRCULATIONAHA.117.028911 (PMC5757664; doi:10.1161/CIRCULATIONAHA.117.028911)

SUPPLEMENTAL MATERIAL

Requests by researchers to access the data, analytic methods, and study materials for the purposes of reproducing the results or replicating procedures can be made to the corresponding author who manages the information.

Coronary artery ligation

Mice aged 10–12 wk (25–30 g) were anesthetized by inhalation of 4% isofluorane gas (Isoflo, Abbott Laboratories, USA) delivered in O_2 at 1.5 L/min. Animals were given preoperative analgesia of 5 mg/kg carprofen (Rimadyl, Pfizer Animal Health, U.) and 0.1 mg/kg buprenorphine (Vetergesic, Reckitt Benckiser Healthcare Ltd, UK) i.p. delivered in 0.4 mL of sterile saline. Mice were endotracheally intubated and maintained with artificial ventilation at 120 breaths/min with a tidal volume of 120 µL on 1% isofluorane in 0.5 L/min O_2. Mice were positioned in a left lateral oblique position. The skin was incised perpendicular to the sternum in parallel with the ribs 5 mm proximal to the xyphoid process. Underlying muscles were carefully retracted to expose the fifth intercostal space beneath. The intercostal muscle was cut using an electrocautery pen and the ribs retracted to expose the heart. The pericardial sac was opened and removed to provide access to the left anterior descending (LAD) coronary artery which was then ligated with 0.3 metric nylon (W2829 Ethilon, Johnson & Johnson, UK) 1.5 mm distal to the left atrial appendage. Three sutures were evenly pre-placed along the ribs using 0.7 metric non-absorbable prolene (W8711, Johnson & Johnson, UK). The lungs were reinflated until the pre-placed sutures were tied. The thoracic muscles were returned to their original position and skin closed with 0.7 metric absorbable vicryl (W9575, Johnson & Johnson, UK). Animals that underwent the sham procedure had a thoracotomy without LAD ligation. Temporary LAD ligation (I/R model) was achieved by using a piece of polyethylene tubing (PE-10; outer diameter 0.61 mm) against which the coronary artery was tied temporarily (45 min) and then subsequently released by removal of the tubing. Post-operatively, animals were given buprenorphine orally for 3 days.

RNA isolation, cDNA synthesis and real-time qPCR analysis

Total RNA was extracted from heart tissue using the miRNeasy Mini Kit (Qiagen, UK) with DNase I treatment (Qiagen, UK). 1 µg of RNA was then reverse transcribed to cDNA with Omniscript reverse transcriptase, dNTPs, RNase inhibitors and oligo dT primers (Omniscipt Reverse Transcription kit, Qiagen, UK). Real-time quantitative PCR (qPCR) reactions were run using cDNA with a Runx1 quantitect primer assay (Amplicon length-120bp, Mm_Runx1_1_SG QuantiTect Primer Assay [QT00100380], Qiagen, UK) and SYBR green mastermix (Applied Biosystems, UK) or with a Runx1 Taqman Gene Expression Assay (Amplicon length 81bp, Mm01213404_m1) and Taqman Univeral Mastermix Mix II, no UNG (both ThermoFisher Scientific, UK) in a 20 µL final volume or 10 µL final volume respectively with the following cycling conditions: hold for 2 min at 50°C followed by 10 min at 95°C to heat-start the *Taq* polymerase enzyme, then 40 cycles of 95°C for 10 min, 60°C for 1 min. Relative mRNA levels were analysed using comparative Ct calculations; either $2^{-\Delta\Delta Ct}$ (regional comparisons relative to RV region) or $2^{-\Delta Ct}$ (whole heart or cardiomyocytes) normalised to Gapdh (whole heart; Eurofins MWG Operon, Germany), or Gapdh Taqman Gene Expression Assays (Amplicon length 109 Mm99999915_g1, ThermoFisher Scientific, UK) or Peptidylprolyl Isomerase B; PPIB (isolated cardiomyocytes).

Generation of mice with conditional cardiomyocyte-specific gene excision of *Runx1*

We generated cardiomyocyte-specific *Runx1*-deficient mice using standard Cre-LoxP-based gene targeting strategies (Supplemental Material Figure 5). This approach utilized mice with LoxP sites flanking exon 4 of the *Runx1* gene (*Runx1*$^{fl/fl}$) and mice with the cardiac-specific alpha-myosin heavy chain (MHC) promoter directing expression of a tamoxifen-inducible Cre recombinase (MerCreMer) to adult cardiomyocytes[1]. Cardiomyocyte-specific excision of *Runx1* was induced in adult mice by a single i.p. injection of tamoxifen (40 mg/Kg) to prevent cardiac dysfunction arising from repeated injections[2].

Genomic PCR was performed to determine the specificity of the *Runx1* gene disruption 7 d after injection with tamoxifen (Supplemental Material Figure 5A–C). Excision of exon 4 (denoted by the PCR product presenting at approximately 310 bp following gel eletrophoresis) was clearly present in all male and female mice. However, this size PCR product was absent in control *Runx1$^{fl/fl}$* mice, which instead produced only a 275 bp PCR product, denoting insertion of the LoxP sites. Control *Runx1$^{wt/wt}$* mice exhibited only a 203 bp PCR product representing an unaltered *Runx1* gene as expected. To determine the extent to which the *Runx1* gene had been excised from cardiomyocytes, PCR of genomic DNA was performed on both: (i) whole hearts and (ii) cardiomyocytes that had been separated from other cell types by plating and short term culture or by FACS (Supplemental Material Figure 5C). Isolated cardiomyocytes exhibited an increased ratio of the excised PCR product (310 bp) to the combined excised (310 bp) and LoxP product (275 bp) indicating approximately ~50% *Runx1* gene excision (Supplemental Material Figure 5C).

Whilst the mRNA *Runx1* levels were significantly reduced in whole heart tissue taken from the right (RV) and left ventricle (LV) of *Runx1$^{Δ/Δ}$* mice compared to control mice (*Runx1$^{wt/wt}$* and *Runx1$^{fl/fl}$*) 4 wk post-MI, this was not the case in the border zone (BZ) and infarct (INF) regions reflecting the increased number of non-cardiomyocytes in these regions post-MI (Supplemental Material Figure 5D).

At 2 wk post-MI, mRNA *Runx1* levels (relative to the house keeper gene Peptidylprolyl Isomerase B; PPIB) in cardiomyocytes isolated from *Runx1$^{Δ/Δ}$* mice were 61% of the control mice (*Runx1$^{fl/fl}$*) (0.055±0.006 vs. 0.090±0.012 ($2^{-Δct}$); *P*<0.05; Supplemental Material Figure 5E) and Runx1 protein levels in cardiomyocytes isolated from *Runx1$^{Δ/Δ}$* mice were 48% of the control mice (*Runx1$^{wt/wt}$* and *Runx1$^{fl/fl}$*) (47.6±9.6 vs. 100±3.84% change; *P*<0.05; Supplemental Material Figure 5F) indicating that protein and mRNA expression correlates with the level of gene excision in these cells.

PCR of genomic DNA

Mice were killed by the schedule one procedure. Hearts were removed and perfused via the aorta with saline solution to wash out the blood before being snap frozen in liquid N_2 and stored at –80˚C. The tissue was homogenized with the TissueLyser system (QIAGEN) using a stainless steel bead in each sample. Tissue was disrupted mechanically in proprietary lysis buffer (Illustra Nucelon Genomic DNA Extraction kit, GE Healthcare) until no visible pieces of tissue remained (~2 min at 25 oscillations/s). Lysates were incubated for at least 3 h with proteinase K at 50˚C. DNA was extracted according to the manufacturer's instructions. Primers used were as described by Chen *et al.* [3]: Runx1 forward primer WT and floxed alleles (Ex4Int – F563) 5'- CCC ACT GTG TGC ATT CCA GAT TGG -3'; Runx1 reverse primer for WT and floxed alleles (Ex4 – R837) 5'- GAC GGT GAT GGT CAG AGT GAA GC -3'; and Runx1 reverse primer for deleted floxed allele (Int3-2) 5'- CAC CAT AGC TTC TGG GTG CAG -3'. Herculase II Fusion DNA polymerase (Agilent) was used under the following stepwise cycling conditions:

1 95°C 1 min

2 95°C 20 s

3 58°C 20 s

4 68°C 1 min

5 Repeat steps 2–4, 30 times

6 68°C 4 min

7 12°C hold

RNAscope® assay

RNA *in situ* hybridization was performed using the RNAscope® duplex kit (Red/Brown) (Advanced Cell Diagnostics) on the Leica Bond Rx Autostainer to detect Runx1 and PCM-1 using mouse-specific probes (Advanced Cell Diagnostics) on formalin fixed paraffin embedded (FFPE) mouse hearts. PCM1 has been shown to be 95-99.9% specific to cardiomyocytes furthermore, cardiomyocytes could be identified morphometrically by their

elongated nucleus and/or the presence of surrounding muscle striations[4-6]. Adult mouse hearts at 1 and 14 day post-MI and SHAM FFPE were cut at 4 μm and placed in an oven 60°C for 1 h. The duplex staining protocol was performed following the manufacturer's (Advanced Cell Diagnostics) strict guidelines. For each heart, positive (PPIB and POLR2A) and negative controls (bacterial dapB) were run (Supplemental Material Figure 1). The images were visualized on a brightfield Evos Cell Imaging microscope. Cells were considered to be positive if at least one brown (PCM-1) or red (Runx1) punctate dot was present within the nuclei - a single punctate dot representing a single mRNA transcript[7]. Cells were manually counted for quantification as previously described[7] taking three representative images per region of the heart on mid-heart sections where the aorta is confluent with the LV chamber.

Pressure–volume loop measurements

Pressure–volume (PV) loop measurements were recorded using the Transonic ADV500 small animal model PV measurement system or a Scisense PV loop system and a 1.2 F 4.5 mm spaced PV admittance catheter (FTH-1212B-4517, Scisense, UK) as previously described[8]. Mice were anesthetized in a pre-filled induction chamber with 4% isofluorane gas (Isoflo, Abbott Laboratories, USA) delivered in O_2 at 1.5 L/min. Mice were endotracheally intubated and maintained with artificial ventilation at 120 breaths/min with a tidal volume of 120 μL. Isofluorane concentration was gradually reduced to 1.5% during the procedure and maintained at this level for the duration of the measurements. The temperature was maintained at 37ºC by a homeothermic monitoring system (Harvard Apparatus, UK). A longitudinal incision was made in the center of the neck and the right carotid artery dissected free of the surrounding tissue. A small incision was made in the vessel and the catheter inserted into the vessel and advanced into the left ventricle of the heart. Steady state readings were then taken for 15 min. Data were recorded in the Labscribe software *via* a four-channel analog-to-digital converter. Data were analyzed offline by averaging the final 2 min of steady state recording.

Echocardiography

Mice were anesthetized as for pressure–volume loop measurements and maintained *via* facemask on 0.5-1% isofluorane in 1.0 L/min O_2.

Histology

Hearts from killed animals were fixed for a minimum of 24 h in 10% neutral buffered formalin (CellPath*,* UK) after which time they were embedded into a wax block for sectioning. Each heart was sliced parallel to the long axis every 200 μm to produce 4 μm serial sections. At every 200-μm interval, picrosirius red was used to stain collagen until the mid-point depth of the heart (defined by the largest ventricular cavity size and confluence of the LV chamber with the aorta). Quantification of regional areas and infarct size was performed on each section using Image J and Adobe Photoshop CC 2015 and mean data per heart were generated as described in previous studies[9]. The point at which the infarct region became 50% of muscle and 50% of collagen (as determined using picrosirius red staining) was denoted as the BZ in histological sections. Infarct thickness was defined and measured as the distance between the endocardium and epicardium of the infarcted myocardium. This was performed using three lines drawn perpendicular to the curvature of the ventricular wall and the distance measured with ImageJ on each histological section. Cardiomyocyte size was assessed by staining adjacent sections at the level of the middle of the heart with AlexaFluor-594 conjugated wheat germ agglutinin (WGA; Invitrogen, Paisley, UK). Briefly, de-waxed and rehydrated sections were boiled in sodium citrate buffer for 10 min, followed by blocking in 1% BSA/PBS with 5% goat serum for 1 h. Sections were then incubated with 10 μg/mL WGA for 1 hr at room temperature in the dark. Sections were mounted in ProLong Gold with DAPI (Invitrogen, Paisley, UK). Confocal imaging was used to produce an image of the LV and septum from each heart. A digital grid was placed on the image using ImageJ and grid intersections determined which cardiomyocytes where then chosen for measurement of cell length, diameter (perpendicular to centre of longest axis) and cross-sectional area (thus avoiding cell selection bias).

Infarct size at 24 h post-MI and I/R injury.

Infarct size in the mouse MI model was determined using triphenyltetrazolium chloride (TTC) (dissolved at 1% in a phosphate buffer for 15 min [37°C]) to detect viable tissue in x5 transversely cut myocardial slices per heart. In the mouse I/R model, the area at risk (AAR) and infarct size was measured using previously published protocols[10] whereby 1% Evans blue delineated the area not at risk, TTC the viable tissue and infarct region white. Heart slices were then placed into 10% neutral buffered formalin for 20 min at room temperature. Digital photographs of the myocardial sections from each heart were laid onto a white background and whole photograph white balanced against the background using Adobe photoshop. The number of pixels of each region was counted using Image J (National Institute of Health, Maryland, USA) and expressed as relative %.

Adult cardiomyocyte isolation

Hearts were removed and the coronary arteries perfused via the aorta at 4.0 mL/min (37°C) with a Modified Isolation Krebs–Henseleit (MIKH) solution for 4 min. The composition of MIKH (in mmol/L) was: NaCl (120.00), KCl (5.40), HEPES (20.00), NaH_2PO_4 (0.52), $MgCl_26H_2O$ (3.50), taurine (20.00), creatine (10.00), glucose (11.10). The pH was adjusted to 7.4 with NaOH. Perfusion of hearts with MIKH was followed by perfusion with MIKH containing 1.0 mg/mL of type I collagenase (Worthington Biochemical) and 0.1 mg/mL of type XIV protease (Sigma-Aldrich). After ~6 min, the enzymes were removed and the heart perfused with MIKH containing 0.7% BSA (Sigma-Aldrich) but no enzymes for a further 4 min. The left ventricular free wall was then cut into strips and mixed to yield a single-cell suspension in MIKH containing 0.7% BSA. The calcium concentration in solution ($[Ca^{2+}]_o$) was raised in this suspension via stepwise increments until 1.0 mmol/L was reached.

Immunoblotting

Isolated cardiomyocytes were lysed in radioimmunoprecipitation assay (RIPA) buffer. The composition of the RIPA buffer (in mmol/L) was: Tris (20), NaCl (150), EDTA (5), EGTA (5),

DTT (1) plus 1.0% Triton X-100 and 0.5% deoxycholate. MI tissue samples in Supplemental Material Figure 2 were disrupted in extraction buffer (0.1 M Tris–HCl, 0.01 M EDTA, 0.04 M DTT, 10% SDS, pH 8.0) using an ultrasonic device. Protease inhibitors added (in mmol/L) were: Na pyrophosphate (2.5), β-glycerophosphate (1.0), Na_3VO_4 (1.0), PMSF (1.0), NaF (2.0), plus 10 μg leupeptin, one tablet of a protease inhibitor cocktail (Complete Mini, Roche, Germany) and one tablet of phosphatase inhibitor (Phos-stop, Roche, Germany) per 10 mL buffer. The buffer was adjusted to pH 7.4. Lysates were assayed for protein concentration using the bicinchoninic acid assay. BSA was used to produce a standard curve. Samples were mixed with β-mercaptoethanol as the reducing agent and a loading dye before heating at 99˚C for 90 s. The lysates were loaded at a concentration of 10 μg per well into 4–12% Bis-tris gels (NuPAGE, Life Technologies, UK) with 4 μg of lysed thymus tissue as a positive control. Electrophoresis conditions were 75 V for 10 min followed by 165 V for 60–90 min. Protein was transferred to 0.45 μm pore size nitrocellulose membranes (Life technologies, UK).

Membranes were incubated overnight at 4˚C with primary antibodies against: PLB A2 (1:1000; MA3-922, Pierce), Runx1 (1:500; ab35962, AbCam), p-PLB Ser16 and p-PLB Thr17 (1:1000; A010-12 and A010-13, Badrilla), PKC α (1:200; sc-8393, Santa Cruz), PP1 (1:200; sc-7482, Santa Cruz). Pan-actin (1:1000; 4968s, Cell Signalling) or Red Alert Western blot stain (Merck Millipore) were used as loading controls. Secondary antibodies were donkey anti-mouse IRDye 800 CW and donkey anti-rabbit IRDye 680RD (1:10000; 926-32212 and 926-68073, Li-Cor). Western blots were visualized and quantified using a LI-COR fluorescence imager and LI-COR or Quantity One analysis software. Figure 5A, 5D and 5G are separate blots but have been cut and probed separately for each different target. Same lanes were used for $Runx1^{wt/wt}$ MI mice in Figure 5D hence the actin signal is common to both PKC and PLB.

Epifluorescence measurements of field stimulated calcium transients

Isolated cardiomyocytes (1.8 mmol/L $[Ca^{2+}]_o$) were loaded with calcium–sensitive fluorophore (5.0 μmol/L Fura-4F AM, Invitrogen). Cardiomyocytes were incubated in MIKH for 30 min for de-esterification in a cell bath (Cell Microcontrols) followed by superfusion with MIKH at 37°C and field–stimulation (1.0 Hz, 2.0 ms duration, stimulation voltage set to 1.5 x threshold). Caffeine (10 mmol/L, 20 s; without field stimulation) was applied before the protocol. Field-stimulation was started after 10 s of perfusion with MIKH and sustained for 120 s before application of a second caffeine bolus at the end of the protocol. The Fura-4F fluorescence ratio (340/380nm excitation; $R_{340/380nm}$) was measured using a spinning wheel spectrophotometer (Cairn Research Ltd.; sampling rate of 5.0 kHz) to measure the intra-cardiomyocyte $[Ca^{2+}]_i$. Cell-edge detection (IonOptix) was used to measure cell length. Data were analyzed offline. The mean Fura-4F fluorescence ratio was obtained by averaging 12 steady state transients (Origin) and converted to $[Ca^{2+}]_i$ as previously described[11]. Particular experiments utilized pretreated (30 min) and perfusion with the PKA inhibitor H89 (1 μmol/L; Tocris Biosciences, Bristol UK) as previously described[12].

Determination of the in vivo electrocardiogram (ECG) in *Runx1*$^{Δ/Δ}$ and control mice 2 wk-post-MI.

Mice were anaesthetised and maintained at 1 % isoflurane in 1 L/min O_2. ECG was recorded with an IX-228/S data acquisition unit and LabScribe2 software (iWorx) using 3 electrodes, placed subcutaneously in the right forelimb, left forelimb and right hindlimb. The ECG was recorded for 5 min, traces analysed with *Labscribe3* software (iWorx) and the last 20 s of the traces were used to determine the PR and QT intervals, averaging every 30 beats. The QT interval was corrected for heart rate (QT_c), calculated using the QT and RR intervals with the Bazett's formula, adjusted for mice: $QT_c = QT/(RR/100)^{0.5}$.

The QT interval (which coincides well with the action potential duration; APD[13]) increased between sham and MI mice in both groups as expected[13], but there was no significant

difference in QT interval between control and *Runx1*$^{\Delta/\Delta}$ mice post-MI (Supplemental Material Figure 9A&B).

Determination of the action potential duration in cardiomyocytes isolated from *Runx1*$^{\Delta/\Delta}$ and control hearts 2 wk-post-MI.

Voltage recordings were made on isolated cardiomyocytes using the CellOPTIQ™ electrophysiology platform (Clyde Bioscience Ltd., Glasgow, UK). Cells were loaded with 8 µmol/L Di-4 ANEPPS, and illuminated using a 470nm OptoLED (Cairn Research; Faversham, UK). The APD was measured at a range of lengths (APD_{20}, $_{40}$, $_{60}$ and $_{80}$) and demonstrated no significant difference between control and *Runx1*$^{\Delta/\Delta}$ mice post-MI. Together with the ECG measurements above, the experiments demonstrated that an increased APD cannot account for the changes in Ca^{2+} handling between control and *Runx1*$^{\Delta/\Delta}$ mice post-MI (Supplemental Material Figure 9C&D).

Determination of calcium entry in cardiomyocytes isolated from *Runx1*$^{\Delta/\Delta}$ and control hearts 2 wk-post-MI.

Fura-4F (8 µmol/L)-loaded cardiomyocytes were alternately excited between 360 and 380nm with emission collected at 510nm and electrically paced using field stimulation (2ms, 40V, 1Hz). Two previously published protocols were used to measure the amplitude of the Ca^{2+} transient in the absence of SR Ca^{2+} release (an index of Ca^{2+} influx via the L-type Ca^{2+} channel). The first protocol measured the amplitude of the first stimulated Ca^{2+} transient immediately after application of 10mM caffeine (to empty Ca^{2+} from the sarcoplasmic reticulum; SR)[14, 15] (Supplemental Material Figure 9E&F) and in separate experiments, the second protocol measured the amplitude of the stimulated Ca^{2+} transient during inhibition of the SR with thapsigargin (1µmol/L for 30 min; Supplemental Material Figure 9G)[16]. Both protocols confirmed that there was no change in the amplitude of Ca^{2+} influx between control and *Runx1*$^{\Delta/\Delta}$ mice post-MI. Collectively, these experiments

demonstrated that an increased Ca^{2+} entry cannot account for the changes in Ca^{2+} handling between control and *Runx1*$^{\Delta/\Delta}$ mice post-MI.

Viral overexpression of Runx1 in culture

Adult rabbit cardiomyocytes were isolated as described above but in sterile filtered isolation MIKH. The $[Ca^{2+}]$ in solution ($[Ca^{2+}]_o$) was raised in this suspension via stepwise increments until 1.0 mmol/L was reached. Cardiomyocytes were gently centrifuged in a hand-operated centrifuge and resuspended in prewarmed minimal essential media (MEM; Life Technologies) supplemented with L-glutamine and penicillin–streptomycin plus 10% fetal calf serum (FCS). The cells were added to wells of a 6-well tissue culture plate at a density of 1 x 10^5 rods per well and incubated at 37˚C in 5% CO_2 for 1–2 h to allow the cardiomyocytes to adhere to the base of the well. The media was carefully aspirated under sterile conditions. Freshly warmed MEM without FCS was added to each well. Adenovirus was previously prepared with cloned genes for GFP (control Ad-GFP) or Runx1 with GFP (experimental Ad-Runx1) using standard protocols[17, 18]. Aliquots of each virus were thawed and diluted to 1:100 with warmed MEM. The PFU of each virus was 2.54 x 10^{10} for Ad-Runx1 and 1.10 x 10^{11} for Ad-GFP. The volume of the virus required for infection was calculated based on the number of rod-shaped cardiomyocytes and the PFU of the virus. Cultures were maintained at 37˚C in 5% CO_2 for 24 h.

REFERENCES

1. Sohal DS, Nghiem M, Crackower MA, Witt SA, Kimball TR, Tymitz KM, Penninger JM and Molkentin JD. Temporally regulated and tissue-specific gene manipulations in the adult and embryonic heart using a tamoxifen-inducible Cre protein. *Circ Res*. 2001;89:20-25.

2. Hall ME, Smith G, Hall JE and Stec DE. Systolic dysfunction in cardiac-specific ligand-inducible MerCreMer transgenic mice. *Am J Physiol Heart and Circ Physiol*. 2011;301:H253-60.

3. Chen MJ, Yokomizo T, Zeigler BM, Dzierzak E and Speck NA. Runx1 is required for the endothelial to haematopoietic cell transition but not thereafter. *Nature*. 2009;457:887-91.

4. Gilsbach R, Preissl S, Gruning BA, Schnick T, Burger L, Benes V, Wurch A, Bonisch U, Gunther S, Backofen R, Fleischmann BK, Schubeler D and Hein L. Dynamic DNA methylation orchestrates cardiomyocyte development, maturation and disease. *Nat Commun*. 2014;5:5288.

5. Bergmann O, Zdunek S, Alkass K, Druid H, Bernard S and Frisen J. Identification of cardiomyocyte nuclei and assessment of ploidy for the analysis of cell turnover. *Exp Cell Res*. 2011;317:188-94.

6. Preissl S, Schwaderer M, Raulf A, Hesse M, Gruning BA, Kobele C, Backofen R, Fleischmann BK, Hein L and Gilsbach R. Deciphering the Epigenetic Code of Cardiac Myocyte Transcription. *Circ Res*. 2015;117:413-23.

7. Wang F, Flanagan J, Su N, Wang LC, Bui S, Nielson A, Wu X, Vo HT, Ma XJ and Luo Y. RNAscope: a novel in situ RNA analysis platform for formalin-fixed, paraffin-embedded tissues. *J Mol Diagn*. 2012;14:22-9.

8. Clark JE and Marber MS. Advancements in pressure-volume catheter technology - stress remodelling after infarction. *Exp Physiol*. 2013;98:614-21.

9. Takagawa J, Zhang Y, Wong ML, Sievers RE, Kapasi NK, Wang Y, Yeghiazarians Y, Lee RJ, Grossman W and Springer ML. Myocardial infarct size measurement in the mouse chronic infarction model: comparison of area- and length-based approaches. *J Appl Physiol (1985)*. 2007;102:2104-11.

10. Bohl S, Medway DJ, Schulz-Menger J, Schneider JE, Neubauer S and Lygate CA. Refined approach for quantification of in vivo ischemia-reperfusion injury in the mouse heart. *Am J Physiol Heart Circ Physiol*. 2009;297:H2054-H2058.

11. Elliott EB, Hasumi H, Otani N, Matsuda T, Matsuda R, Kaneko N, Smith GL and Loughrey CM. K201 (JTV-519) alters the spatiotemporal properties of diastolic Ca^{2+} release and the associated diastolic contraction during beta-adrenergic stimulation in rat ventricular cardiomyocytes. *Basic Res Cardiol*. 2011;106:1009-22.

12. Zhang M, Prosser BL, Bamboye MA, Gondim AN, Santos CX, Martin D, Ghigo A, Perino A, Brewer AC, Ward CW, Hirsch E, Lederer WJ and Shah AM. Contractile Function During Angiotensin-II Activation: Increased Nox2 Activity Modulates Cardiac Calcium Handling via Phospholamban Phosphorylation. *J Am Coll Cardiol*. 2015;66:261-72.

13. Boukens BJ, Rivaud MR, Rentschler S and Coronel R. Misinterpretation of the mouse ECG: 'musing the waves of Mus musculus'. *J Physiol*. 2014;592:4613-26.

14. Elliott EB, McCarroll D, Hasumi H, Welsh CE, Panissidi AA, Jones NG, Rossor CL, Tait A, Smith GL, Mottram JC, Morrison LJ and Loughrey CM. Trypanosoma brucei cathepsin-L increases arrhythmogenic sarcoplasmic reticulum-mediated calcium release in rat cardiomyocytes. *Cardiovasc Res*. 2013;100:325-35.

15. Trafford AW, Diaz ME, Negretti N and Eisner DA. Enhanced Ca^{2+} current and decreased Ca^{2+} efflux restore sarcoplasmic reticulum Ca^{2+} content after depletion. *Circ Res*. 1997;81:477-484.

16. Elliott EB, Kelly A, Smith GL and Loughrey CM. Isolated rabbit working heart function during progressive inhibition of myocardial SERCA activity. *Circ Res*. 2012;110:1618-27.

17. Theriault FM, Nuthall HN, Dong Z, Lo R, Barnabe-Heider F, Miller FD and Stifani S. Role for Runx1 in the Proliferation and Neuronal Differentiation of Selected Progenitor Cells in the Mammalian Nervous System. *J Neurosci*. 2005;25:2050-2061.

18. Nicklin SA and Baker AH. Simple methods for preparing recombinant adenoviruses for high-efficiency transduction of vascular cells. *Methods Mol Med*. 1999;30:271-83.

Supplemental Material Figure 1. Control RNAscope probes. Typical control heart section images of **(A)** negative dapB and **(B)** positive PPIB and POLR2 probes taken from the right ventricle (RV), left ventricle (LV), border zone (BZ) and infarct (INF) regions in MI hearts 1 day post-MI or the equivalent regions in sham hearts.

Supplemental Material Figure 2. Runx1 expression in WT C57BL/6 mice post-MI. (A) Typical PV loop traces at 4 weeks post-MI and mean data (*n*=10 sham, *n*=13 MI; **P*<0.05,

Student's t-test) for; **(B)** Left ventricular (LV) End-systolic pressure, **(C)** maximum rate of contraction, **(D)** LV end-diastolic pressure, **(E)** maximum rate of relaxation, **(F)** LV end-systolic volume, **(G)** LV end-diastolic volume and **(H)** Ejection fraction. **(I)** Whole heart *Runx1* mRNA (*n*=6 sham, *n*=7 MI; **P*<0.05, Student's t-test). **(J)** MI heart (BZ,border zone; LV,left ventricle; INF,infarct; RV,right ventricle; Scale=1mm). **(K)** Regional *Runx1* mRNA 4 wk post-MI (*n*=4 sham, *n*=8 MI). #*P*<0.05 relative to RV and **P*<0.05 sham versus MI, Student's t-test. (RQ,relative quantification). **(L)** Regional Runx1 protein 3 wk post-MI (*n*=5), with **(M)** mean data (ANOVA).

Supplemental Material Figure 3. Regional *Runx1* mRNA 8 wk post-MI

Runx1 mRNA in different regions (INF, infarct; BZ, border zone and LV, left ventricle) of 8 wk post-MI hearts (*n*=4 sham, *n*=6 MI; **P*<0.05 Student's t-test).

Supplemental Material Figure 4. Echocardiography study – cardiac contractile function in C57Bl/6J mice. 1 wk post-MI echocardiography study measuring fractional shortening in WT C57Bl/6J sham (*n*=3-5) and MI (*n*=3-6) mice; **P*<0.05 Student's t-test.

Supplemental Material Figure 5. Generation of *Runx1*$^{\Delta/\Delta}$ mice. (A) The RUNT domain and locations of the three primers (colored arrows) used to detect the *Runx1*$^{wt/wt}$, *Runx1*$^{fl/fl}$ and excised *Runx1*$^{\Delta/\Delta}$ alleles. LoxP sites are indicated by blue triangles. **(B)** PCR of genomic DNA; whole hearts from male (M) and female (F) mice (*Runx1*$^{\Delta/\Delta}$ *n*=9 for M and *n*=4 for F; *Runx1*$^{fl/fl}$ *n*=8 for M and *n*=6 for F; *Runx1*$^{wt/wt}$ *n*=6 for M and *n*=6 for F). A WT and three-band control where used to delineate the appropriate sized bands (bands 1 and 2). **(C)** PCR of genomic DNA; WT and three-band controls (bands 1 and 2), whole heart preparations (bands 3–5), cardiomyocytes (CMs) from *Runx1*$^{\Delta/\Delta}$ mice that had been separated from other cell types by plating and short term culture (band 6) or by FACS (band 7). Mean ± SEM of *Runx1* excision as denoted by the ratio of the excised band (310 bp) to the combined excised (310 bp) and LoxP band (275 bp) (band 5 [*n*=3], band [*n*=4)] and band 7 [*n*=2]),

*$P<0.05$ ANOVA. **(D)** *Runx1* mRNA levels (relative to PPIB; $2^{-\Delta Ct}$) in whole heart tissue from the right ventricle (RV), left ventricle (LV), border zone (BZ) and infarct (INF) regions of 4 wk post-MI hearts (*Runx1*$^{\Delta/\Delta}$ MI [n=5 hearts] as % of *Runx1*$^{wt/wt}$ and *Runx1*$^{fl/fl}$ MI [n=9 hearts] *$P<0.05$ Student's t-test). **(E)** *Runx1* mRNA levels (relative to PPIB; $2^{-\Delta Ct}$) in cardiomyocytes separated from other cell types by plating and short term culture from 2 wk post-MI hearts (*Runx1*$^{\Delta/\Delta}$ MI [n=7 hearts] as % of *Runx1*$^{fl/fl}$ MI [n=8 hearts], *$P<0.05$ Student's t-test). **(F; Left)** Western blot of Runx1 in isolated cardiomyocytes from 2 wk post-MI hearts with pan-actin loading control (performed on same blot as Figure 6A) and thymus (Thy) positive control for Runx1. **(F; Right)** Percentage change in Runx1 protein in isolated cardiomyocytes from 2 wk post-MI hearts (*Runx1*$^{\Delta/\Delta}$ MI [n=5 hearts] as % of *Runx1*$^{wt/wt}$ and *Runx1*$^{fl/fl}$ MI [n=5 hearts], *$P<0.05$ Student's t-test).

Supplemental Material Figure 6. Blinded study – cardiac function assessed by echocardiography in *Runx1*$^{\Delta/\Delta}$ mice. 2 wk post-MI blinded study (control *Runx1*$^{fl/fl}$ MI (n=8), *Runx1*$^{wt/wt}$ MI (n=11) and *Runx1*$^{\Delta/\Delta}$ MI (n=7), *$P<0.05$ ANOVA) for the following functional parameters: **(A)** Fractional shortening (FS), **(B)** Left ventricular internal diameter at systole (LVIDs), **(C)** LVID at diastole (LVIDd), **(D)** LV posterior wall thickness at systole (LVPWs), **(E)** LVPW thickness at diastole (LVPWd).

Supplemental Material Figure 7. Echocardiography study – cardiac contractile function in *Runx1*$^{\Delta/\Delta}$ mice. 1 wk post-MI echocardiography study measuring fractional shortening in *Runx1*$^{\Delta/\Delta}$ MI (n=5-6) and control *Runx1*$^{fl/fl}$ MI and *Runx1*$^{wt/wt}$ MI mice combined (n=5-10); *$P<0.05$ Student's t-test.

Supplemental Material Figure 8. Infarct size at 24 h post-myocardial infarction (MI) and post-ischemia reperfusion (I/R). (A) Typical heart section images of MI using TTC staining. **(B)** Mean infarct area (IA) as % of left ventricle (LV) [control MI (n=8) and *Runx1*$^{\Delta/\Delta}$

MI (*n*=5), **P*<0.05 Student's t-test]. **(C)** Typical heart section images of IR using TTC staining. **(D)** Mean area at risk (AAR) as % LV (left) and IA as % of AAR (right) [control MI (*n*=6) and *Runx1*$^{Δ/Δ}$ MI (*n*=4), **P*<0.05 Student's t-test]. Scale bar: 1mm Linear adjustment of contrast, brightness or color were applied equally to all parts of the image.

Supplemental Material Figure 9. Isolated cardiomyocyte electrical activity in *Runx1*$^{Δ/Δ}$ mice. (A) Typical electrocardiogram (ECG) traces from control and *Runx1*$^{Δ/Δ}$ and mice 2 wk-post-MI with **(B)** mean QT and QT_c (corrected) intervals of control *Runx1*$^{fl/fl}$ and *Runx1*$^{wt/wt}$ mice combined (*n*=4 sham; *n*=10 MI) and *Runx1*$^{Δ/Δ}$ (*n*=4 sham; *n*=6 MI), **P*<0.05 Student's t-test. **(C)** Typical voltage recordings on isolated cardiomyocytes from control and *Runx1*$^{Δ/Δ}$ and mice 2 wk-post-MI with **(D)** mean action potential duration (APD) at a range of lengths (APD_{20}, $_{40}$, $_{60}$ and $_{80}$) of control *Runx1*$^{fl/fl}$ MI and *Runx1*$^{wt/wt}$ MI mice combined (*n*=28 cardiomyocytes, 6 hearts) and *Runx1*$^{Δ/Δ}$ MI (*n*=18 cardiomyocytes, 5 hearts). **(E)** Typical calcium measurements of the Ca^{2+} transient in the absence of SR Ca^{2+} release in isolated cardiomyocytes from control and *Runx1*$^{Δ/Δ}$ and mice 2 wk-post-MI with **(F)** mean amplitude of the first stimulated Ca^{2+} transient immediately after application of 10mM caffeine of control *Runx1*$^{fl/fl}$ MI and *Runx1*$^{wt/wt}$ MI mice combined (*n*=31 cardiomyocytes, 7 hearts) and *Runx1*$^{Δ/Δ}$ MI (*n*=11 cardiomyocytes, 3 hearts). **(G)** Mean amplitude of the stimulated Ca^{2+} transient during thapsigargin-mediated SR inhibition of control *Runx1*$^{fl/fl}$ MI and *Runx1*$^{wt/wt}$ MI mice combined (*n*=6 cells; 2 hearts) and *Runx1*$^{Δ/Δ}$ MI (*n*=5 cells; 2 hearts).

SUPPLEMENTAL MATERIAL FIGURE 1

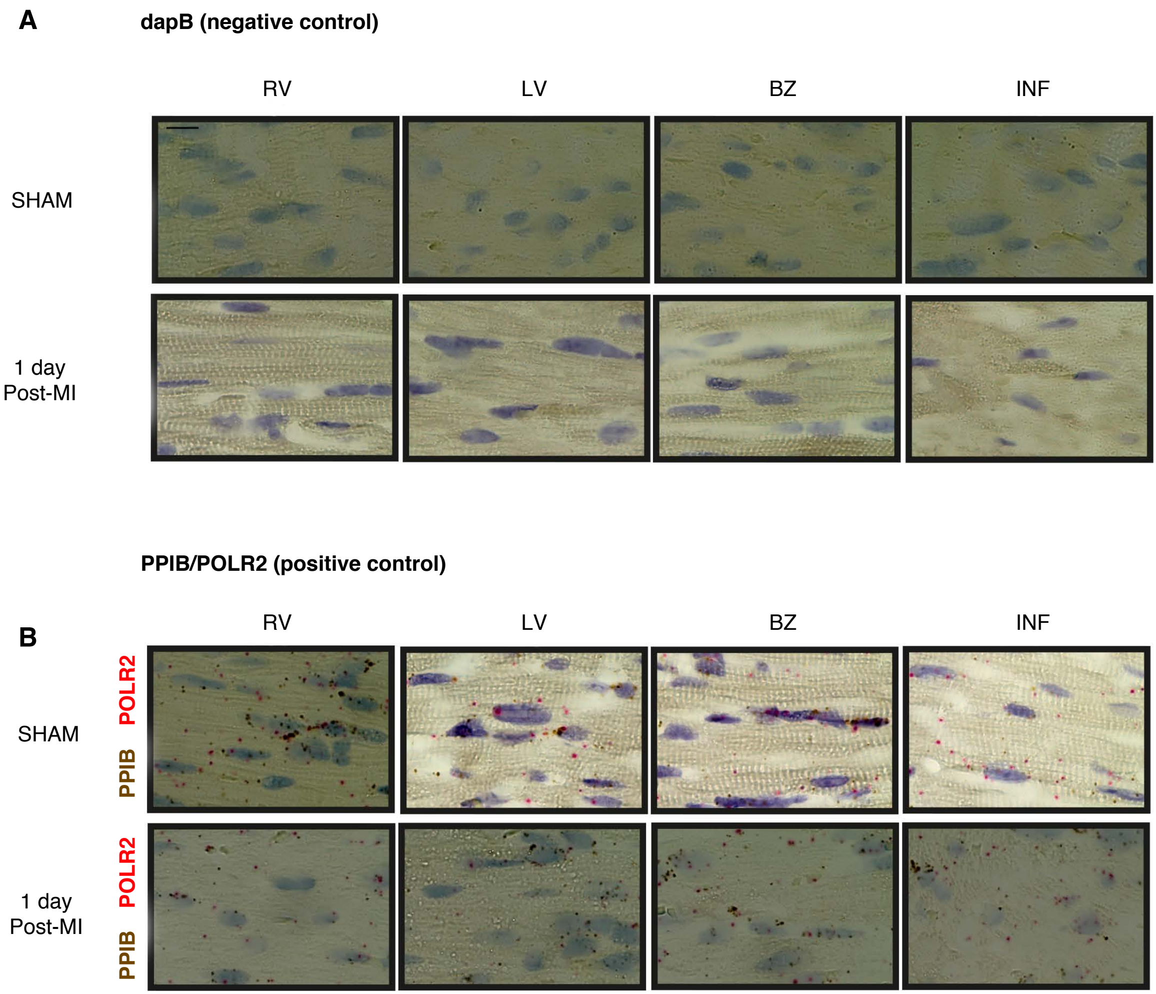

SUPPLEMENTAL MATERIAL FIGURE 2

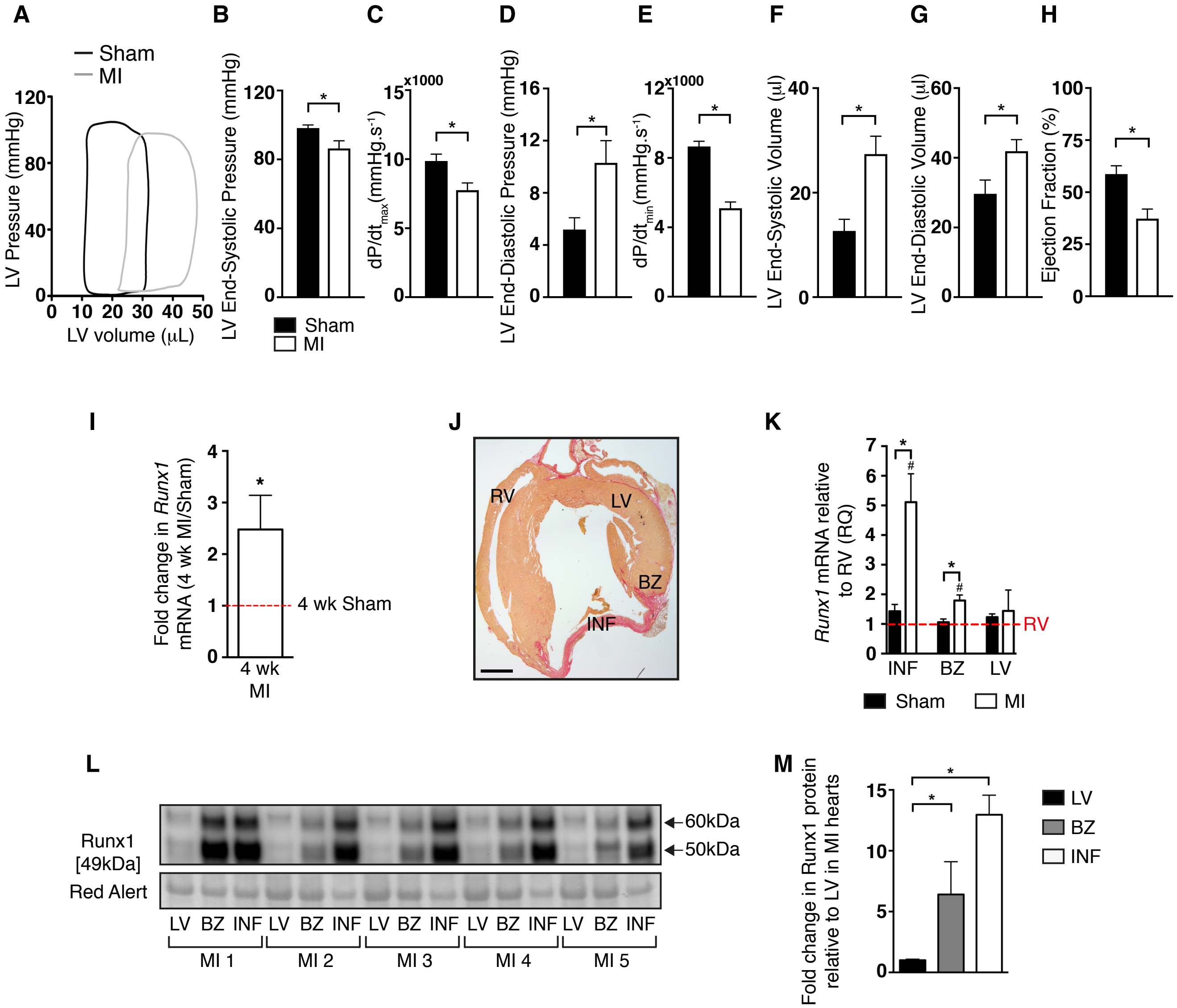

SUPPLEMENTAL MATERIAL FIGURE 3

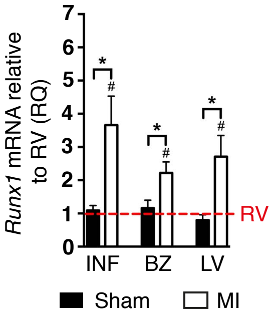

SUPPLEMENTAL MATERIAL FIGURE 4

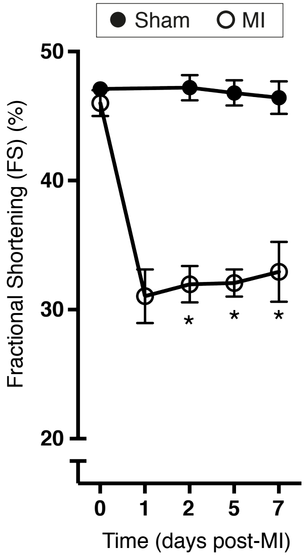

SUPPLEMENTAL MATERIAL FIGURE 5

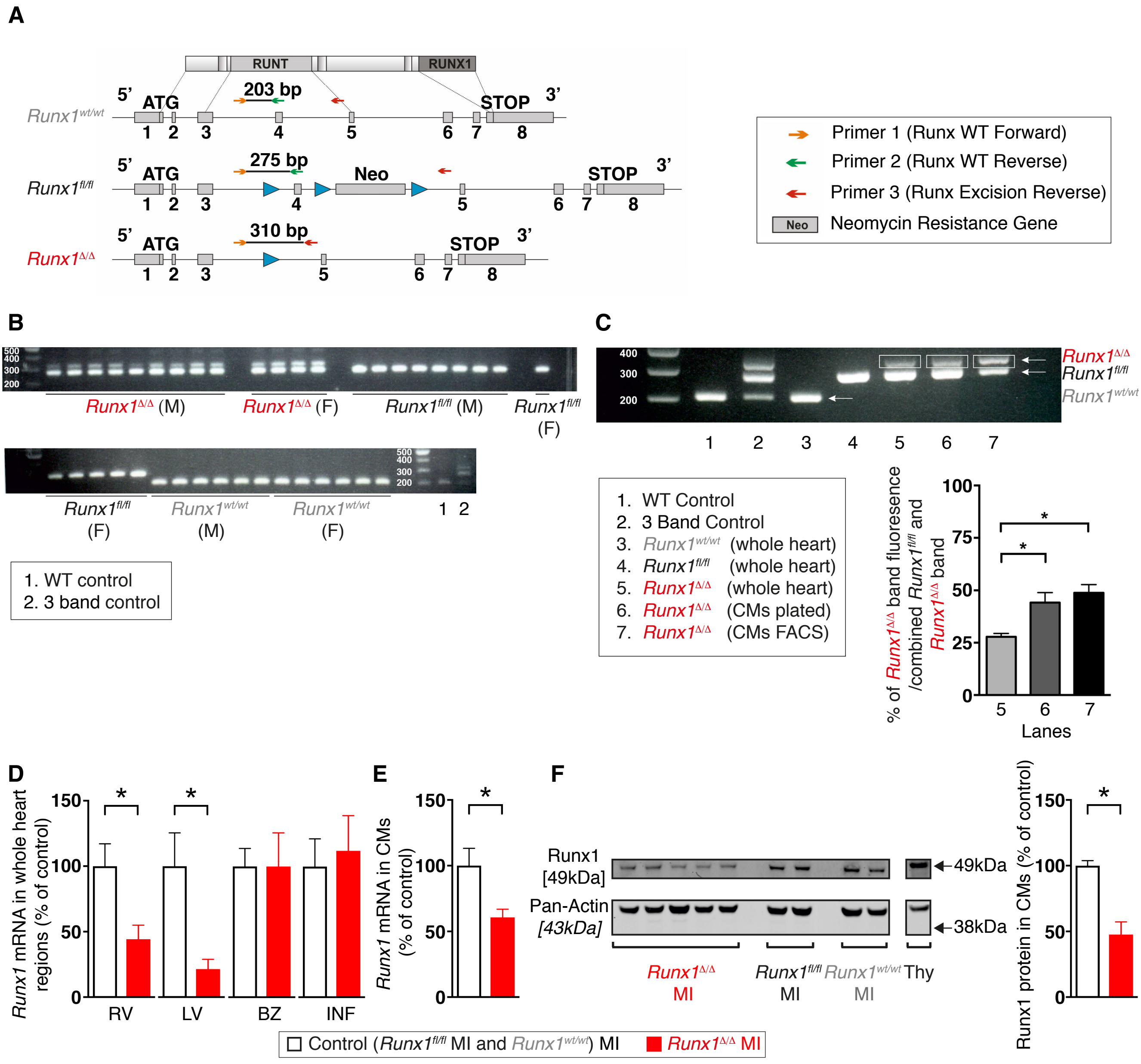

SUPPLEMENTAL MATERIAL FIGURE 6

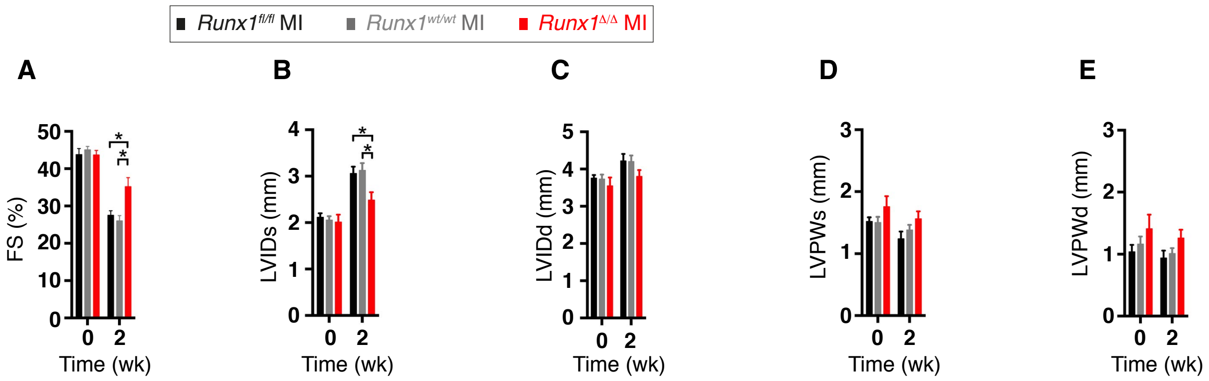

SUPPLEMENTAL MATERIAL FIGURE 7

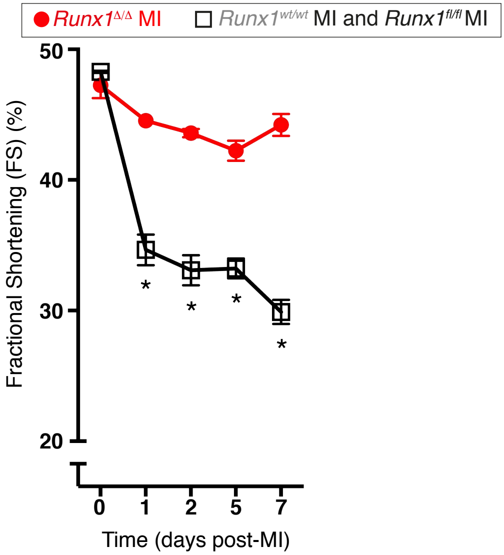

SUPPLEMENTAL MATERIAL FIGURE 8

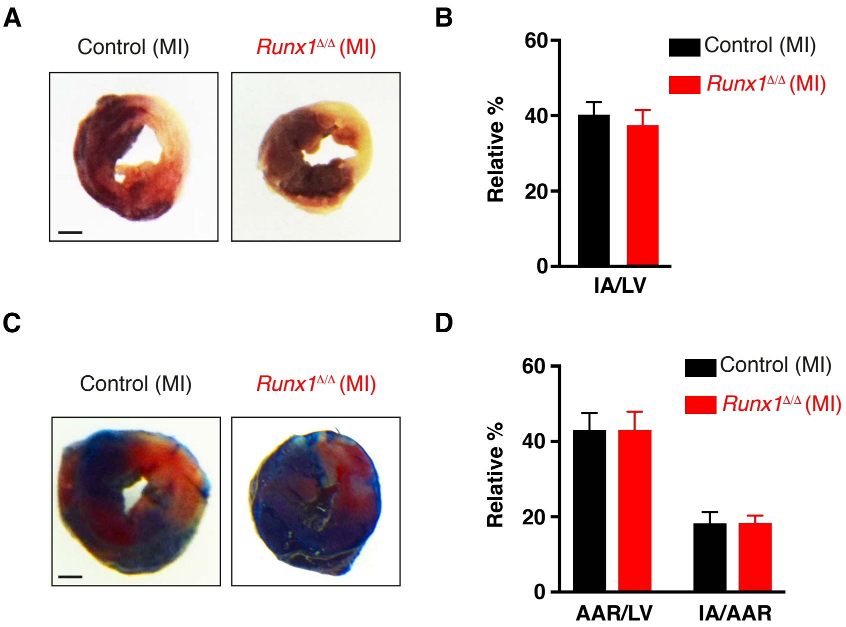

SUPPLEMENTAL MATERIAL FIGURE 9

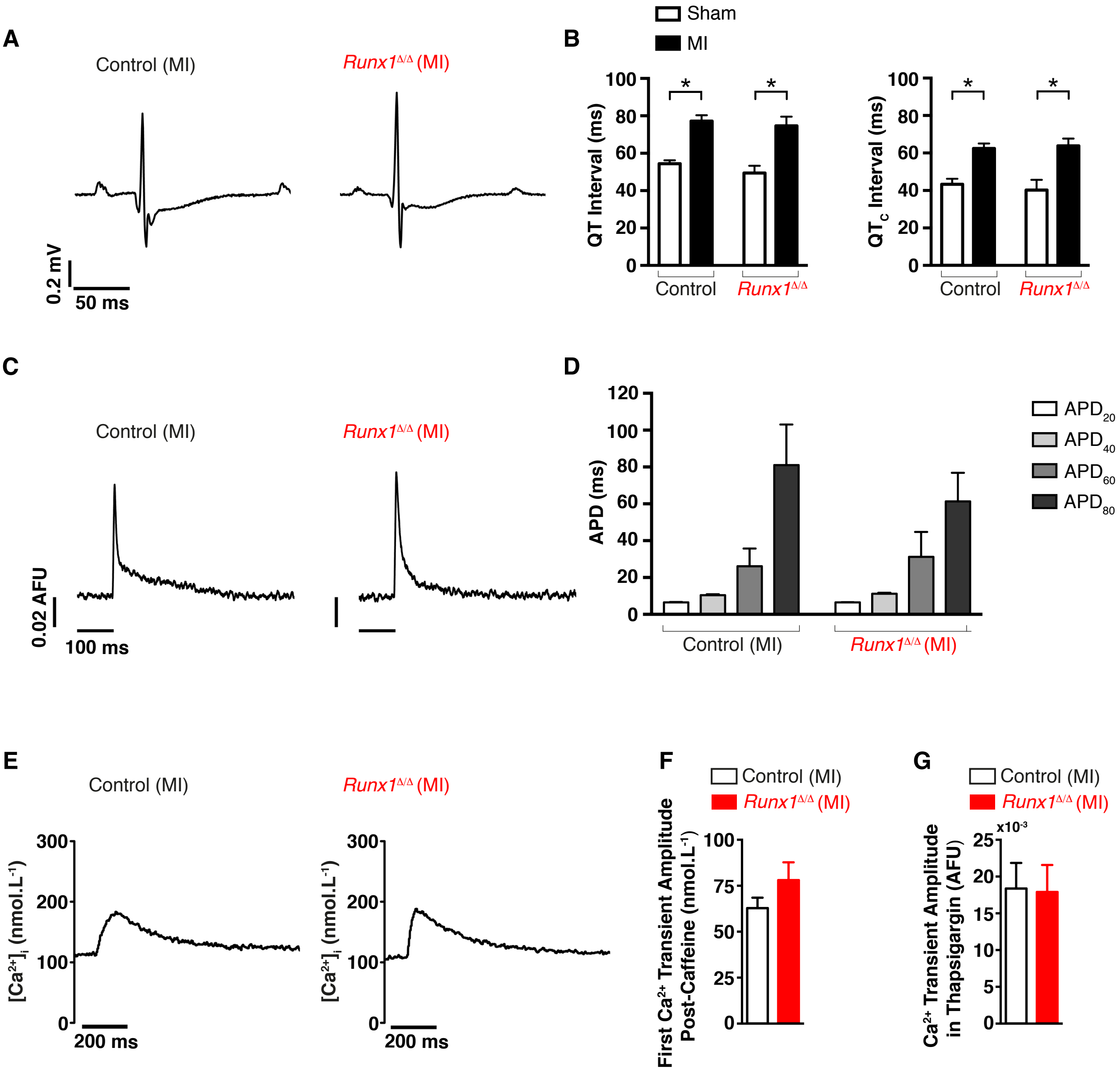

Supplement: Supplementary file 1 [file cir-137-57-s001.pdf]
